# High-Resolution Phenotypic Landscape of the RNA Polymerase II Trigger Loop

**DOI:** 10.1371/journal.pgen.1006321

**Published:** 2016-11-29

**Authors:** Chenxi Qiu, Olivia C. Erinne, Jui M. Dave, Ping Cui, Huiyan Jin, Nandhini Muthukrishnan, Leung K. Tang, Sabareesh Ganesh Babu, Kenny C. Lam, Paul J. Vandeventer, Ralf Strohner, Jan Van den Brulle, Sing-Hoi Sze, Craig D. Kaplan

**Affiliations:** 1 Department of Biochemistry & Biophysics, Texas A&M University, College Station, Texas; 2 Mississippi State University, Starkville, Mississippi; 3 Yale Cardiovascular Research Center, Dept. of Internal Medicine, Yale University School of Medicine, New Haven, Connecticut; 4 Department of Veterinary Pathobiology, Texas A&M University, College Station, Texas; 5 Roche Nimblegen, Madison, Wisconsin; 6 Shire Pharmaceutical, Boston, Massachusetts; 7 College of Medicine, Texas A&M Health Science Center, College Station, Texas; 8 Department of Biochemistry & Molecular Biology, Baylor College of Medicine, Houston, Texas; 9 MorphoSys AG, Planegg, Germany; 10 Department of Computer Science and Engineering, Texas A&M University, College Station, Texas; University of Wisconsin-Madison, UNITED STATES

## Abstract

The active sites of multisubunit RNA polymerases have a “trigger loop” (TL) that multitasks in substrate selection, catalysis, and translocation. To dissect the *Saccharomyces cerevisiae* RNA polymerase II TL at individual-residue resolution, we quantitatively phenotyped nearly all TL single variants *en masse*. Three mutant classes, revealed by phenotypes linked to transcription defects or various stresses, have distinct distributions among TL residues. We find that mutations disrupting an intra-TL hydrophobic pocket, proposed to provide a mechanism for substrate-triggered TL folding through destabilization of a catalytically inactive TL state, confer phenotypes consistent with pocket disruption and increased catalysis. Furthermore, allele-specific genetic interactions among TL and TL-proximal domain residues support the contribution of the funnel and bridge helices (BH) to TL dynamics. Our structural genetics approach incorporates structural and phenotypic data for high-resolution dissection of transcription mechanisms and their evolution, and is readily applicable to other essential yeast proteins.

## Author Summary

Proper regulation of Pol II transcription, the first step of gene expression, is essential for life. Extensive evidence has revealed a widely conserved and dynamic polymerase active site component, termed the Trigger Loop (TL), in balancing transcription rate and fidelity while possibly allowing control of transcription elongation. Coupling high-throughput sequencing with our previously established genetic system, we are able to assess the *in vivo* phenotypes for almost all possible single substitution Pol II TL mutants in the budding yeast *Saccharomyces cerevisiae*. We show that mutants in the TL nucleotide interacting and linker regions widely confer dominant and severe growth defects. Clustering of TL mutants’ transcription-related and general stress phenotypes reveals three main classes of TL mutants, including previously identified fast and slow elongating mutants. Comprehensive analyses of the distribution of fast and slow elongation mutants in light of existing Pol II crystal structures reveal critical regions contributing to proper TL dynamics and function. Evidence is presented linking a previously observed hydrophobic pocket to NTP substrate-induced TL closing, the mechanism critical for correct substrates selection and transcription fidelity. Finally, we assess the functional interplay between TL and its proximal domains, and their presumptive roles in the function and evolution of the TL. Utilizing the Pol II TL as a case study, we present a structural genetics approach that reveals insights into a complex, multi-functional, and essential domain in yeast.

## Introduction

RNA polymerase II (Pol II) synthesizes all eukaryotic mRNAs. Structural studies of *Saccharomyces cerevisiae* (*Sce*) Pol II have illuminated mechanisms of transcription [1–6], especially RNA synthesis. RNA synthesis occurs through iterative nucleotide addition cycles (NACs): selection of correct substrate nucleoside triphosphate (NTP), catalysis of phosphodiester bond formation, and enzyme translocation to the next template position. These critical steps in NAC appear to be coordinated by a critical, conserved domain within the Pol II active site: the trigger loop (TL).

**Fig 1:**
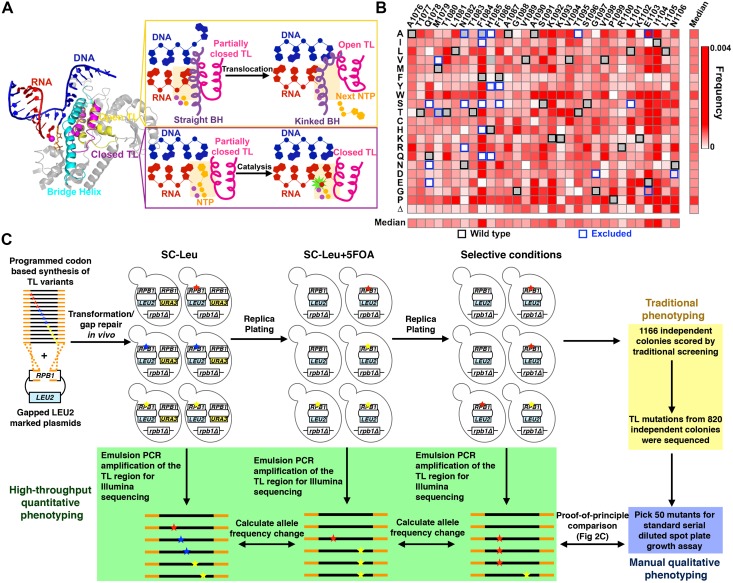
Establishment of a high-throughput platform for phenotyping comprehensive TL single variant library. (A) Multiple TL functions are underpinned by its mobile nature. Structures of open (PDB:5C4J) and closed TLs (PDB:2E2H) are shown in the context of surrounding domains. Template DNA (blue), RNA (red), Bridge Helix (cyan), Closed TL (magenta) and Open TL (yellow) are shown in cartoon rendering. The open TL has been proposed to allow Pol II translocation while the closed TL has been shown to facilitate catalysis (right panel). (B) Mutational coverage of the TL variant library is shown as a heatmap illustrating the allele frequencies of single substitution variants (WT amino acids and positions labeled on x axis; amino acid substitutions on y axis). The WT amino acids for each position are noted with black boxes, and mutants excluded from library synthesis are noted using blue boxes. (C) Schematic representation of experimental approach. Stars of different colors represent distinct substitutions. The TL variant library PCR amplicon (encoding Rpb1 amino acids 1076–1106) flanked by *RPB1 TL* flanking sequence (orange) was co-transformed with a linearized *LEU2 CEN* plasmid containing an *rpb1* gene with the TL deleted, allowing construction of full-length *RPB1* (with TL variants) by *in vivo* homologous recombination. Heterozygous Leu^+^ transformants were replica-plated onto SC-Leu+5FOA to select against the WT *RPB1* (*URA3 CEN*) plasmid and to create TL variant pools. TL variant pools were subsequently replica-plated to different selective conditions for either traditional individual colony screening or high-throughput phenotyping using deep sequencing. For the latter, replica-plated colonies were pooled for genomic DNA extraction, and the TL region was amplified by emulsion PCR to prepare templates for deep sequencing.

TL functions are underpinned by its mobile and flexible nature (Fig 1A). The primary function of the TL is kinetic selection of correct NTP substrates while balancing transcription speed and fidelity, and this function is highly conserved based on studies of RNAPs from *Escherichia coli* (*Eco*) [7,8], *Thermus aquaticus* (*Taq*) [9], the archaeons *Pyrococcus furiosus* (*Pfu*) [10] and *Methanocaldococcus jannaschii* (*Mja*) [11], and eukaryotic Pol II from *Sce* [12,13] and human [14]. In a simplified two-step model, correct NTP binding appears to facilitate TL movement such that a bound, matched NTP shifts the TL from the “open” state to the “closed” state [4,15–18], allowing capture of the matched NTP in the Pol II active site and promotion of phosphodiester bond formation [4,17,19]. The subsequent release of the byproduct, pyrophosphate, allows a conformational shift of the TL from the “closed” state back to the “open” state [15,20,21]. TL opening has been proposed to be critical for enzyme translocation relative to the DNA template, an essential step for the next nucleotide addition cycle [8,13,15,22–25]. Furthermore, additional TL states have been implicated in transcriptional pausing from studies in *E*.*coli* [17,22,26], backtracking from structural observations [27,28], and, although controversial, intrinsic cleavage [7,29–32]. Thus, distinct TL conformations or interactions are linked to different functions in transcription, with delicate control of TL dynamics promoting proper transcription elongation while possibly incorporating signals from the rest of Pol II or Pol II bound factors [17,33–36].

Genetic and biochemical studies have revealed TL functions in the NAC. First, the nucleotide interacting region (NIR, Rpb1 1078–1085) discriminates matched rNTPs from 2’-dNTPs and non-complementary rNTPs [12,13]. NIR substitutions in residues observed to interact with rNTPs widely conferred lethality. Where viable, substitutions reduced catalytic activity *in vitro* and were termed as partially loss-of-function (LOF) [7–10,12,37]. Second, a TL C-terminal mutant E1103G, conferred increased catalytic activity *in vitro*, which we termed gain-of-function (GOF) [12,13,38]. Fast kinetics experiments revealed that E1103G may bias TL dynamics towards the catalytically active “closed” state [13], consistent with infidelity and compromised translocation in addition to increased catalysis [12,13,23,39,40]. Furthermore, we previously described a set of Pol II TL mutants with broad and distinct alterations to transcription *in vivo*, thus conferring allele-specific phenotypes (Table 1) that correlate with decreased or increased activity [37,41] *in vitro*. Various genetic interactions (suppression, exacerbation, and epistasis) have also been observed among TL substitutions, suggesting a complex functional network within the Pol II TL [37]. Finally, context dependence for TL residue function has been observed, wherein analogous mutations in a conserved TL residue showed opposite effects in *Sce* Pol I and Pol II, suggesting different rate limiting steps for the two enzymes [42]. Together, the intricate intra-TL functional network and the context dependence of TL properties suggest importance of the extensive residue-residue interactions within and outside the TL.

**Table 1:** Plate phenotypes employed for the screening Pol II alleles *in vivo*.

Phenotype	Affected Gene/Reporter Allele; Pol II mutant class affected	WT growth	Mutant growth
Sensitivity to 5FOA	Detects ability of *rpb1 LEU2* plasmid to complement *rpb1*Δ [37]	Resistance to drug. *RPB1 LEU2* plasmid complements *rpb1*Δ	Sensitivity to drug (Partial or no complementation of *rpb1*Δ by *rpb1 LEU2)*
Suppressor of Ty (Spt^-^)	*lys2-128*∂; reports on chromatin defects and start site selection [52]. Specific class of GOF Pol II mutants [37,41,53].	Lysine auxotroph (Lys^-^)	Lysine prototroph (Lys^+^)
Mycophenolic acid sensitivity (MPA^S^)	*IMD2* expression required for resistance; reports on start site selection [57,58]. Specific classes of GOF and LOF Pol II mutants [37,41,56].	Resistance to drug	Sensitivity to drug for GOF mutants, relative resistance for LOF mutants.
Modulation of transcriptional readthrough at *gal10*Δ*56* (Gal^R^*)*	*gal10*Δ*56*; likely reports on termination, mRNA processing and initiation [37,41,54,55]. It is found widely in LOF Pol II mutants, some GOF.	Moderate sensitivity to galactose (Gal^S^)	Resistance to galactose (Gal^R^)

The possible multifunctional nature of each TL residue complicates interpretations of functions if interpretations are based on a limited number of mutants. This is because the phenotype of any given mutant could result from removal of the wild type side-chain or additional functions of the substituted residue. Furthermore, different substitutions may have distinct effects on particular TL conformations [37,43]. In the TL, different substitutions in the same residue can confer distinct phenotypes, so limiting mutational analyses to a single substitution at a particular position may mislead about residue function [13,37]. Deep mutational scanning is an emerging technique for studying large sets of mutants by assessing the enrichment or depletion of variants after a strict selection process [44]. Different selection approaches have been designed such that a specific protein property (sensitivity to substitutions [45], thermo-stability [46], protein stability [47], *etc*) can be studied. Notably, our established genetic phenotypes (Table 1) were well correlated with altered transcription elongation rates *in vitro* and specific transcription defects *in vivo* [37,41], thus providing a powerful phenotypic framework for studying TL function. In this work, we have defined the fitness and phenotypic landscape of the conserved, essential *S*. *cerevisiae* Pol II TL. We have found three distinct classes of transcriptionally defective TL mutants that are associated with differential stress response profiles, allowing the determination of functional contributions of each TL residue. We have examined the mechanisms by which proximal Pol II domains communicate with the TL, while identifying examples of inter-residue epistasis, which are the likely drivers of incompatibility of RNAP evolutionary variants when placed in the Pol II context.

## Results

### Strategy for studying *in vivo* effects of TL variant library

**Fig 2:**
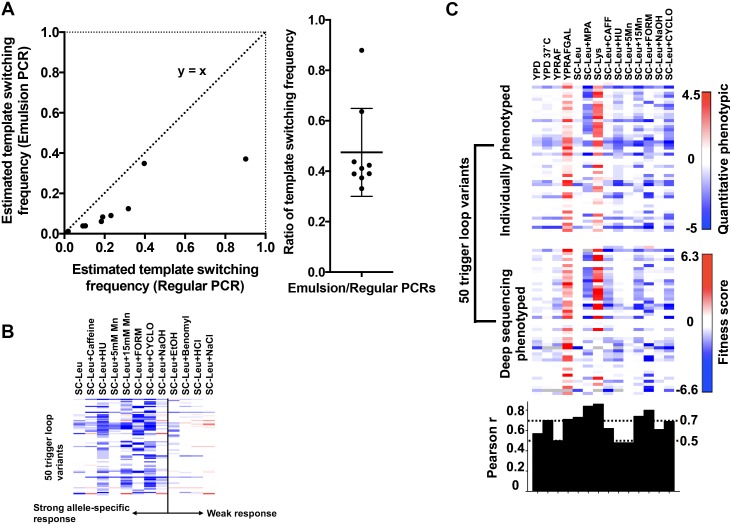
Quality controls for the TL high-throughput phenotyping approach. (A) Comparison of estimated template switching frequencies in regular and emulsion PCR conditions. Template switching was estimated by the ratio (Freq^Double^) / (Freq^Single1^ × Freq^Single2^) for all the possible double mutants combined from five spiked-in single mutants. (B) Additional growth conditions were employed to increase resolution for distinguishing similar TL alleles. Growth scores for 50 individually isolated TL mutants (y axis) under 12 growth conditions (x axis), as determined by standard serial dilution plate phenotyping (S2 and S3 Figs), are shown as a heatmap. Positive values shown in red indicate increase in allele frequency relative to WT and negative values in blue indicate decrease in allele frequency relative to WT. (C) High-throughput quantitative phenotyping results are consistent with individual phenotyping of variants. Top heatmap shows qualitative growth scores (as in Fig 2B) of 50 individually phenotyped TL variants on the y axis (S2–S4 Figs) with selective conditions on the x axis. Deep sequencing results for the same mutants using median of fitness defects from three independent high-throughput screens are shown in the middle panel. Pearson r calculated to show the correlation between each condition from the two datasets is shown in the bottom panel.

A comprehensively mutagenized TL variant library (Rpb1 1076–1106), excepting some previously well-characterized variants [12,37], was synthesized using the Slonomics technology [48,49] and validated by deep sequencing (Fig 1B). Synthesis conditions were such that single substitution mutants would predominate. Our TL mutant library showed an even distribution of substitutions across all positions and substitution types (S1A and S1B Fig), with generally very low frequencies for excluded mutants, as expected (Fig 1B). We first sought evidence that the measured allele frequencies reflected the real allele frequency distribution because PCR fidelity for highly similar amplicons is often compromised by template switching [50,51]. We spiked in five excluded single substitution variants (H1085Y, H1085Q, F1086S, G1097D, E1103G) as controls. Double mutant variants comprised of these single substitution spike-in variants would not be present in our library, but if observed they would presumably be the result of template switching between spike-ins. We prepared TL amplicons from a subset of conditions using both standard PCR and emulsion PCR (emPCR), which can suppress template switching [50,51]. First, double mutants derived from spike in controls were found at a significantly lower frequency than the relevant single substitution variants; Second, emPCR further suppressed the template switching frequency for all possible double mutants derived from spike-in single variants (Fig 2A, left), at about 2.5-fold on average (Fig 2A, right). We conclude that template switching is likely not extensive in our reactions but further reduction by emPCR led us to employ emPCR for our studies.

We have developed an experimental pipeline to examine mutations in an essential gene using a plasmid shuffling strategy, and have applied it to study the TL variant library (Fig 1C). To validate our pipeline and to isolate novel TL alleles, we performed a traditional genetic screening for mutants with transcriptional defects (Table 1). We have shown previously that these phenotypes correlate with Pol II biochemical activity *in vitro* [12,37,41]. Transcription-related phenotypes employed include, first, the Suppressor of Ty (Spt^-^) phenotype, derived from a transposon insertion into the 5′ end of the *LYS2* gene (*lys2-128∂* allele) [52,53]. The transposable element insertion renders wild-type cells Lys^-^. A subset of Pol II TL mutants allow expression of a normally silent promoter within the transposable element to express a truncated but functional *LYS2* transcript, conferring the Spt^-^ phenotype by allowing cells to become Lys^+^. Spt^-^ mutants in the TL correlate with biochemical GOF phenotypes and their related genetic interaction and gene expression signatures [37,41,53]. Second, we employed suppression of the galactose-induced toxicity conferred by the *gal10*Δ*56* allele of *GAL10*, (Gal^R^) [54,55]. *gal10*Δ*56* contains a deletion in the major *GAL10* polyadenylation signal, allowing transcription readthrough and interference with the downstream *GAL7* gene [54,55]. This readthrough/interference alters the ratio of metabolic enzymes in the galactose-utilization pathway, causing the buildup of a toxic intermediate, resulting in galactose sensitivity (Gal^S^). Mutations in transcription elongation factors and Pol II subunits can alter these transcription defects and suppress *gal10*Δ*56* galactose sensitivity [37,41,55]. Third, we employed Mycophenolic acid (MPA) sensitivity. Sensitivity to MPA for examined Pol II TL mutants derives from altered transcription initiation at the *IMD2* promoter [37,56], whose transcription is controlled through use of multiple start sites [57,58], and whose expression is required for cell resistance to MPA [59]. We have linked Pol II catalytic activity to the ability to induce *IMD2*. Increased activity Pol II alleles (GOF) fail to induce *IMD2* in the presence of MPA due to aberrant transcription start site selection [37,56]. By screening for these three transcription-related phenotypes, we isolated 1166 candidate mutants (S1 Table), which included 154 singly-substituted and 386 multiply-substituted variants.

To further distinguish mutants, we examined 50 single substitution variants under various stress conditions to screen for conditions that could induce allele-specific phenotypes (Fig 2B, S2 and S3 Figs). We observed that media containing caffeine, hydroxyurea, MnCl_2_, formamide, cycloheximide, or NaOH induced allele-specific sensitivity or resistance, while media containing ethanol, benomyl, HCl or NaCl showed fewer allele-specific effects (Fig 2B, S2 and S3 Figs). Therefore, in our high-throughput approach, we phenotyped TL variant library under our established conditions (medium lacking lysine (Spt^-^), medium containing MPA (MPA^S^) or medium containing galactose (Gal^R^)) and appropriate media for the stress conditions empirically determined to discriminate among our pilot alleles. Phenotypic scores were estimated from the change of allele frequency normalized to WT, as is standard in mutational scanning studies [44–47]. Quantitative phenotypic scores of the 50 mutants from the high-throughput phenotyping were consistent with semi-quantitative growth scores derived from standard phenotyping (Fig 2C, S2–S4 Figs), validating our approach.

### The Pol II TL fitness landscape

**Fig 3:**
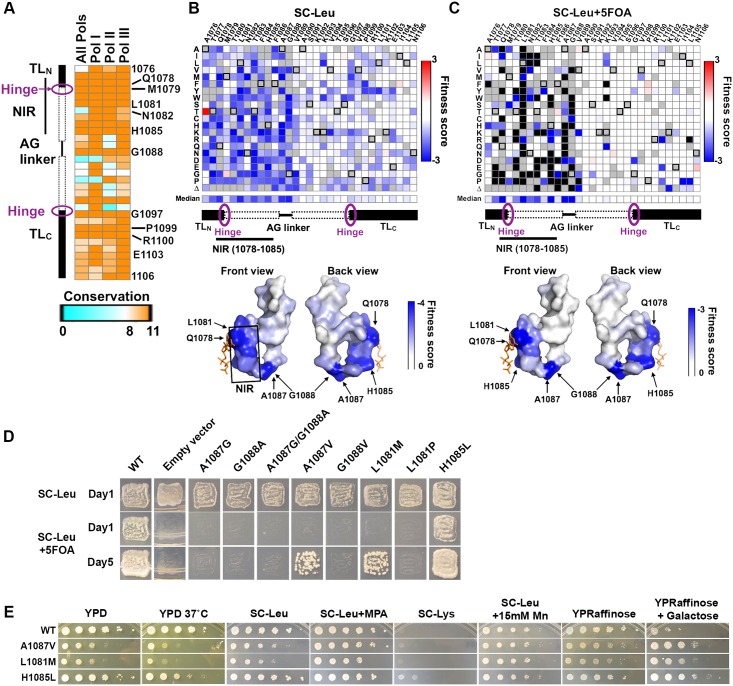
The TL fitness landscape distinguishes highly conserved TL residues and reveals high mutational sensitivity in the nucleotide interacting region (NIR) and the Alanine-Glycine linker. (A) Conservation heatmap of TL residues in eukaryotic RNA polymerases. The conservation scores were extracted from a multiple sequence alignment, including 182 Pol II, 59 Pol I, and 111 Pol III sequences utilizing the conservation metric from Jalview 2.8 version 14.0 [91]. (B) Fitness defects of TL variants in the heterozygous state are shown as a heatmap. Unavailable data points are denoted by filled grey squares. WT residues at indicated positions are denoted by black boxes. Surface representation (bottom panel) of the TL structure (PDB:2E2H) is shaded by the median fitness value for all available variants at each position, in a gradient of white (rare defects) to blue (common defects). The position of the matched GTP substrate is shown in orange stick representation. (C) General fitness defects of TL variants upon removal of WT *RPB1*. Fitness defects predicted to result in lethality shown in black. Surface representation (bottom panel) of the TL structure is shaded by the median fitness value of all available variants at each position, in a gradient of white (rare defects) to blue (common defects). (D) Complementation abilities of variants in the difficult-to-substitute TL positions (L1081, A1087, G1088) or unexpected TL variants (H1085L) assayed by plasmid shuffling of individual strains. Ability to grow on SC-Leu+5FOA indicates complementation of essential functions of *RPB1*. SC-Leu medium is the control state where WT *RPB1* is present. (E) Transcription-linked phenotypes of viable substitutions in difficult-to-substitute residues (L1081M, A1087V) or a TL variant with unexpectedly mild fitness defects (H1085L).

The TL is highly conserved, especially in the NIR, the loop tip residue (Rpb1 G1088) and for several TL C-terminal residues (Fig 3A). Highly-conserved residues are predicted to be critical for protein function, thus substitutions during evolution are expected to confer fitness defects and be selected against. We first sought to evaluate general fitness defects of observed TL singly-substituted variants (termed the “fitness landscape”), both in the presence of WT *RPB1* (Fig 3B) and upon the removal of WT *RPB1* (Fig 3C). Notably, TL NIR and loop tip substitutions conferred large fitness defects in general, while most perturbations in the similarly conserved C-terminal residues did not confer severe growth defects (Fig 3B and 3C). This observation highlights that conservation does not necessarily reflect sensitivity to perturbations, and that the TL fitness landscape can further distinguish extremely highly conserved TL residues, as discussed below:

First, substitutions in the NIR (Rpb1 1077–1085) generally conferred both fitness defects (Fig 3C) and apparent dominance (Fig 3B). Observed fitness defects were consistent with previous observations that several NIR mutants render Pol II slow in elongation *in vitro* and cause fitness defect *in vivo* [12,37]. The observed dominance for many NIR variants was consistent with TL variants being assembled into Pol II complexes that interfere with WT Pol II function, likely through clashes with WT Pol II on genes *in vivo*. Second, substitutions within the alanine-glycine linker (Rpb1 1087–1088) almost universally conferred lethality or severe growth defects. A Pol II structure with a closed TL [4] reveals that A1087 and G1088 are in a tight pocket between the funnel and bridge helices, presumably necessitating small side-chain residues (S5A Fig). To determine the extent of spatial constraint, we individually assessed the fitness of AG swapping variants, and small hydrophobic valine substitutions (Fig 3D). Notably, all the swapping variants (A1087G, G1088A and A1087G/G1088A) were lethal (Fig 3D). While G1088V is lethal, A1087V is severely sick but viable (Fig 3D), suggesting extremely high, but differential spatial constraint but differential tolerability for the two residues. This pocket/TL interaction is only observed in the closed TL [4] but not in any of the open states [60], suggesting function in stabilizing the active, closed TL conformation for promoting catalysis. Consistent with disruption of the pocket/TL interaction and the closed TL state, we observed genetically LOF phenotypes for A1087V (Gal^R^, slight MPA^R^) (Fig 3E). Finally, substitutions in the conserved C-terminal helix, though not strongly defective in general fitness, are likely to have transcription defects, based on our prior studies, and were further characterized (discussed below).

### Novel TL NIR mutants allow mechanistic insights

The TL fitness landscape identified residues highly sensitive to perturbations, while also revealing variants in NIR residues previously known to be difficult to viably substitute. We highlight L1081 and H1085 as two examples. L1081 directly interacts with the nucleobase moieties of matched NTPs [4], and equivalent residues in *Eco*, *Taq* and *Pfu* RNAPs are important for substrate selection or catalysis [7,9,10]. L1081 is the most sensitive residue to perturbations among the hyper-conserved NIR. All previously tested L1081 variants were lethal [37], though viable substitutions were identified for all other NIR residues of interest. Furthermore, the GOF allele E1103G can generally suppress lethal substitutions for most NIR residues, but could not for tested L1081 substitutions [37]. In our TL fitness landscape, almost all L1081 variants were indeed predicted to be lethal based on our fitness threshold (Fig 3C). L1081M conferred a severe growth defect, but was predicted to be just above the viable threshold (Fig 3C). To validate this prediction, we constructed L1081M for direct analysis, and found that L1081M was indeed viable yet severely sick (Fig 3D). Furthermore, L1081M conferred Gal^R^ and slight MPA^R^ phenotypes, consistent with other LOF mutants (Fig 3E). Eukaryotic multi-subunit RNA Polymerases share a stringent evolutionary requirement for L at this TL position, while bacterial and archaeal lineages show both M and L variants. Consistent with evolutionary tolerance of variation within bacterial and archaeal lineages, the *Taq* RNAP M1238L variant shows near WT activity for substrate selection and catalysis *in vitro* [9]. The severe growth defect of L1081M highlights epistasis within *Sce* Pol II and likely eukaryotic RNAP lineages, which imposes a stringent requirement for Leucine at this position.

H1085 interacts with the β-phosphate of the matched NTP [4], and has been implicated in substrate selection, catalysis, intrinsic cleavage and PPi release [29,61]. We previously constructed several H1085 variants (A/N/D/F were lethal, K/R/W/Y caused severe growth defects, Q caused slight growth defect [12,37,41]), suggesting that some polar or positively charged residues, but not a hydrophobic phenylalanine or alanine, could partially complement loss of the histidine [37]. Here, we found that H1085L was viable and healthy in the fitness landscape (Fig 3C), and validated it with phenotypic analyses of a reconstructed H1085L allele (Fig 3D). While H1085L conferred slight MPA^R^ and Gal^R^ phenotypes, consistent with other LOF mutants (Fig 3E), it also conferred a slight Spt^-^ defect, suggesting distinct defects from most other NIR mutants and all known LOF mutants [37]. This observation alters our understanding of the likely bounds of active site chemistry (see discussion).

### There are at least three distinguishable TL mutant classes

The overall TL fitness landscape revealed the essentiality of almost all single substitution TL variants in standard growth medium, but could not indicate the nature of transcriptional defects, as we had previously found that both LOF and GOF alleles conferred growth defects. Therefore, we sought to determine the phenotypic outcome of the TL variants for the transcription-related Gal^R^, MPA^S^ and Spt^-^ phenotypes and a variety of allele-distinguishing stress conditions (investigated earlier in Fig 2A). Here, we term this response profile as the “phenotypic landscape”, as it distinguishes the TL mutants with presumably distinct transcription defects, in contrast to the general “fitness landscape” described above.

**Fig 4:**
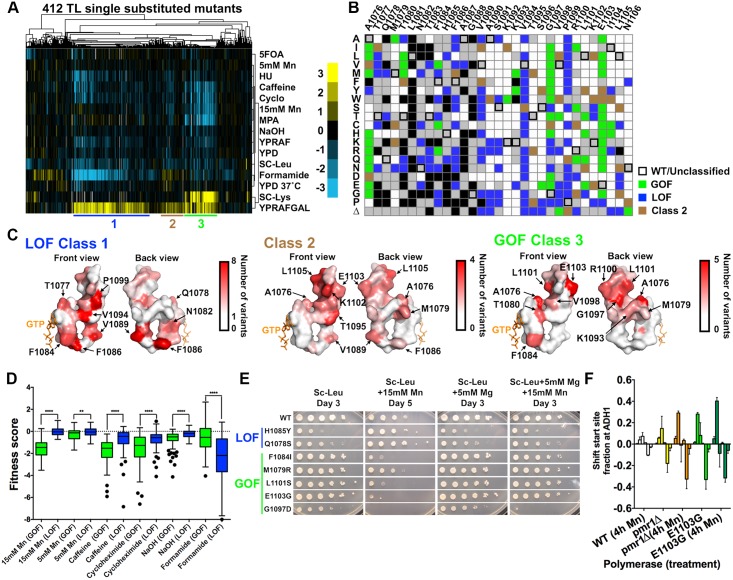
Three distinct TL mutant classes, revealed from TL phenotypic landscape, have specific distribution on the TL structure and distinct stress response profiles. (A) Hierarchical clustering of 412 single TL variants’ (x axis) phenotypes (calculated as in Fig 1C) under 14 different conditions (y axis) reveals distinct mutant classes. Positive (yellow) and negative (blue) fitness scores are shown as a heatmap. Mutant classes (clusters) are annotated by colored lines beneath the heatmap. (B) Distribution of three major mutant classes is shown in a single substitution variant heatmap. Class 1 (genetic GOF) mutants are shown in green; Class 2 mutants are shown in brown and Class 3 (genetic LOF) mutants are shown in blue. (C) Distribution of different mutant classes on the TL structure. TL is shown in surface and colored in the gradient from white to red by the number of clustered mutants at each position. (D) Differential stress responses in genetic GOF and LOF mutants. Genetic GOF mutants are more sensitive to Mn^2+^, caffeine and cycloheximide, whereas genetic LOF mutants are more sensitive to formamide. **p<0.01, ****p<0.0001 (Two-tailed unpaired t-test). (E) Differential Mn^2+^ sensitivity and its suppression by Mg^2+^ for selected TL variants representative of mutant classes. (F) Mn^2+^ effects on different mutants’ transcription start sites (TSSs) distribution at *ADH1*, determined by primer extension analysis. TSSs at *ADH1* are distributed in a range of positions and were divided into six bins for quantitation: from upstream (left) to downstream (right). Change of TSSs (normalized to untreated WT) is calculated by the change in TSS fraction for each bin relative to the WT distribution. Average and standard deviation of three experimental replicates are shown as a bar graph with error bars.

Hierarchical clustering of the phenotypic landscape for 412 TL variants passing fitness filters revealed three major mutant classes with distinct features (Fig 4A and 4B). Class 1 mutants generally conferred a strong Gal^R^ phenotype yet were Spt^+^, and in some cases were also slightly MPA^R^ relative to WT, consistent with previously characterized LOF mutants. We also identified high formamide sensitivity as a new signature phenotype for Class 1 mutants. Class 2 mutants showed generally weaker Gal^R^, slight formamide resistance, and did not confer strong phenotypes otherwise, representing a novel TL mutant class yet to be biochemically characterized. Class 3 mutants generally conferred Gal^R^, Spt^-^ and MPA^S^ phenotypes, consistent with previously characterized GOF mutants. Mn^2+^ hypersensitivity (Mn^S^) was correlated broadly with Spt^-^ and MPA^S^ phenotypes, suggesting a relationship among these phenotypes, and consistent with previous *in vitro* biochemical and *in vivo* phenotypic data for a subset of known GOF mutants [62,63]. Notably, our spike-in LOF (F1086S, H1085Q and H1085Y) and GOF mutants (E1103G and G1097D) co-clustered with Class 1 and Class 3 mutants, respectively.

### Functional contribution of TL residues in different states and substrate-induced TL closing mechanism

The distributions within different mutant classes predict distinct functional contributions of TL residues to TL dynamics. Perturbations predicted to bias the TL towards the active, closed TL state have been shown to result in GOF, whereas destabilization of the closed TL state generally leads to LOF [8,12,13,17,37]. Therefore, distributions of Class 1 (LOF) and Class 3 (GOF) mutants predict alterations to TL dynamics, as follows:

Class 1 (LOF) mutants included most variants from F1086, V1089, V1094 and P1099 (Fig 4C, left), suggesting important functions of these residues in stabilizing the closed TL. F1086 and V1089 are both proximal to multiple funnel helix residues when TL is closed [4,18], while F1086 was proposed to orient H1085 for correct substrate interaction [18]. Therefore, alteration of these interactions may disrupt the closed TL state and result in LOF. Alternatively, recent Pol II structures with open TL revealed potential function of F1086-V1089 interaction in TL closing dynamics (S5B Fig) [60]. V1089 forms a backbone-backbone hydrogen bond with F1086 when TL is open, while its side chain flips towards the F1086 to form a hydrophobic interaction when TL is partially closed, suggesting that this side-chain interaction may be important for particular TL states (S5B Fig), though it was not discussed in previous molecular dynamics (MD) studies [18]. Furthermore, V1094 was observed to be proximal to the BH residue K830 in the closed TL state [4]. An interaction between K830 and V1094 side-chains could be counter-intuitive and possibly undervalued. However, neutralization of lysine’s positive charge through ionic interactions (such as D836) can promote hydrophobicity of the lysine side chain [64], supporting the observed K830-V1094 interactions in the TL closed state (S5C Fig). Most variants in V1094 are LOF (Fig 4B), consistent with disruption of K830-V1094 interaction and concomitant destabilization of the closed, active TL conformation.

**Fig 5:**
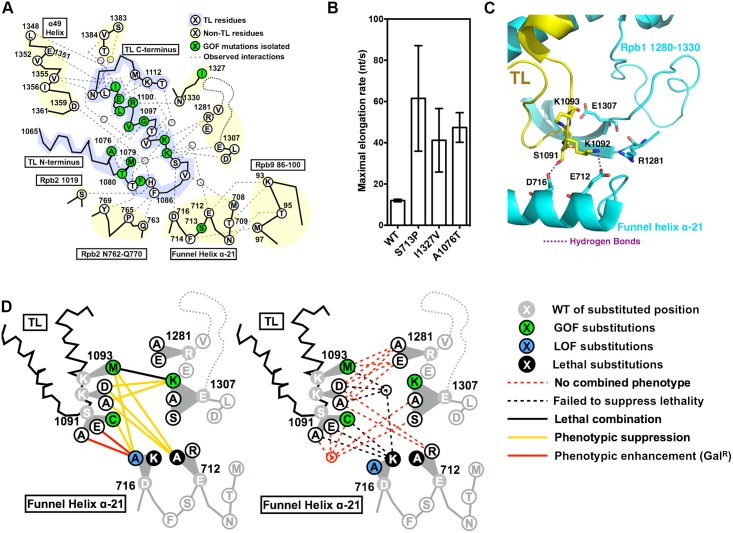
Functional contribution of TL tip and Funnel Helix α-21 to proper TL dynamics. (A) Observed and predicted interactions between TL and TL-proximal domains. TL schematic is shown with residues identified by single-letter amino acid code and positions of interest annotated. Positions of GOF mutants isolated in our screen, along with the positions for a subset of previously isolated TL-proximal GOF mutants, are color coded in green. Observed TL interactions with other Rpb1 domains from structures or simulation studies are shown as grey dashed lines. (B) Maximal *in vitro* elongation rates (nucleotides/second) of Pol II WT and genetic GOF mutants S713P, I1327V and A1076T. (C) Observed interactions between open TL tip and TL adjacent charged residues (PDB: 5C4X). Funnel Helix refers to the Rpb1 α-21 alpha-helix. (D) Genetic interactions between the TL tip and proximal Rpb1 domains. Schematics of the TL and adjacent domains are shown in lines, with positions of interest shown in single-letter amino acid code. Substituted residues are shown in grey, with substituting amino acids shown in white, blue or green filled circles based on single substitution phenotypes (S8F Fig). Double substitution phenotypes are shown as colored lines connecting the two relevant single substitutions. Some sets of similar interactions were grouped into nodes to reduce complexity in interaction lines.

Models for NTP substrate-induced TL closing remain largely untested [4,15–18]. A recent Pol II structure [60] exhibiting an open TL state led to explicit implication of a hydrophobic pocket formed by TL residues (A1076, M1079, T1080, G1097 and L1101) and other TL proximal residues (I837, L841, V1352, V1355 and I1356) in substrate-induced TL-folding (S5D Fig). Q1078 recognition of the 2’-OH of a matched NTP substrate was proposed to promote release of the adjacent residue M1079 from the hydrophobic pocket, triggering TL closing [60,65]. Consistent with disruption of this observed pocket and concomitant destabilization of the inactive open TL state, A1076T, a pocket variant previously isolated as genetically GOF, conferred increased transcription activity *in vitro* (Fig 5B). Notably, GOF phenotypes were observed for a large number of variants in pocket residues. Among them, we observed almost universal GOF phenotypes for G1097 variants, but not the extreme fitness defects found for the previously observed GOF variant G1097D. We individually phenotyped ten G1097 variants from the traditional screening and confirmed this observation (S5E Fig). Together, these results are consistent with the hydrophobic pocket stabilizing the inactive, open TL and providing a plausible mechanism for substrate-induced TL closing. A single residue, M1079, can act as a linchpin for the entire TL through a network of interactions.

### Identification of stress conditions that alter transcription *in vivo*

GOF and LOF TL variant classes have distinct phenotypic profiles. In general, compared to LOF variants, GOF mutants are more sensitive to Mn^2+^, caffeine and cycloheximide yet generally resistant to hydroxyurea and formamide (Fig 4D). The allele-specific Mn^2+^ response amplified our previous observation that the GOF allele E1103G was highly sensitive to Mn^2+^ while the LOF allele H1085Y was resistant to, or even slightly suppressed by, Mn^2+^ (while the Mn^2+^ effects on both mutants were suppressed by Mg^2+^ supplementation) [61]. The TL phenotypic landscape showed that this Mn^2+^ response was general and class-specific for GOF and LOF mutants (Fig 4D). To validate this observation, we individually analyzed seven additional variants (two LOF and five GOF) for Mn^2+^ sensitivity in the presence or absence of Mg^2+^ supplementation. Notably, all tested LOF mutants conferred Mn^2+^ resistance while all tested GOF mutants conferred Mn^2+^ hypersensitivity (Fig 4E). Allele-specific Mn^2+^ responses could be suppressed by Mg^2+^ supplementation (Fig 4E). Mn^2+^ has been shown to stimulate transcriptional activity while compromising fidelity *in vitro* [62,63]. Our observations suggested that Mn^2+^ may suppress LOF mutants by stimulating transcriptional activity yet exacerbate GOF mutants by further decreasing their already compromised transcriptional fidelity *in vivo* [12,13]. Increased Pol II catalytic activity correlates strongly with upstream transcription start site (TSS) shifts *in vivo* [37,41]; therefore we assayed for TSS alterations upon Mn^2+^ treatment. Primer extension analysis at *ADH1* revealed that Mn^2+^ treatment shifted the TSS distribution upstream, and further exacerbated the upstream shift conferred by E1103G (Fig 4F). Deletion of *PMR1*, the golgi Mn^2+^ export channel, causes accumulation cytosolic Mn^2+^ [66,67], and can be used to alter Mn^2+^ levels apart from supplementation of the medium. Our prior high throughput genetic interaction analyses of Pol II mutants showed that *pmr1*Δ strongly interacts with Pol II mutants in a highly allele-specific fashion [41], suggesting an intimate relationship between increased cellular Mn^2+^ levels and altered transcription activity. Here we find that *pmr1*Δ also shifted *ADH1* TSSs upstream (Fig 4F). While Mn^2+^ may have other indirect effects on Pol II mutants, these observations support direct effects of Mn^2+^ on Pol II transcription activity *in vivo*, raising the possibility that other allele-specific stress conditions (*e*.*g*. formamide) may also directly alter transcription *in vivo*.

### Functional contributions of the TL tip region

The TL tip region (Rpb1 1090–1096) is a random-coil region that forms an α-helical structure when the TL is closed, and helical formation has been proposed to assist TL closing [8,18,43]. Mejia *et al* characterized two *Eco* RNAP TL tip mutants I1134V and G1136S (Equivalent to *Sce* Pol II V1094 and S1096) with decreased or increased transcription activity, respectively [43]. These results were interpreted as I1134V and G1136S substitutions decreasing or increasing helical propensity and thus disfavoring or favoring TL closing [43]. *Sce* Pol II contains each of these variants as the WT residue, therefore individual substitutions to the *E*. *coli* variants (V1094I and S1096G) would be predicted to confer opposite phenotypes under the helical propensity model. However, V1094I and S1096G did not confer phenotypes clearly consistent with either GOF or LOF (Fig 4B), failing to support the helical propensity model. We asked if the proposed correlation from *Eco* RNAP studies was a general property for TL substitutions in this region, if extended to more than two substitutions. Our data, calculated from 122 variants, fail to support a general correlation between helical propensity and predicted catalytic activity for Pol II substitutions in this region (S8A Fig). As discussed above, V1094 may be involved in interaction with BH residue K830, and LOF in most V1094 variants may result from disrupted BH/TL coordination. Therefore, we repeated the analyses excluding V1094 variants, yet still failed to observe a correlation (S8A Fig). We cannot rule out contributions of helical propensity in this region to TL function; however, we did not find compelling or widespread evidence for it.

A number of recent studies have suggested potential functions of the TL tip region in regulating TL dynamics [18,60,68]. In a simulated TL closing process, positively charged K1092 and K1093 were predicted to interact with several TL-proximal residues, and some of the predicted interactions were validated by subsequent Pol II crystal structures with alternative open TL states (Fig 5A). These interactions were proposed to stabilize the open, inactive TL state, and thus alanine (K1092A, K1093A) or charge reversing substitutions (K1092D/E, K1093D/E) were predicted to disrupt the inactive TL open state and result in GOF [18]. Contrary to this prediction, none of the above substitutions conferred GOF (Fig 4B). Networks of residue-residue interactions near the TL tip were observed [18,60], some of which may be functionally overlapping or redundant, adding complexity to simple models. Our previous point mutant epistatic miniarray profile (p-EMAP) studies predicted two TL-proximal mutants (S713P and I1327V) to be GOF, which we confirm here (Fig 5B), suggesting that perturbation near the TL may interfere with native interactions, or create new ones, to destabilize the open TL. The tested variants here also extend the correlation between genetically predicted GOF and increased activity *in vitro* (Fig 5B). Additionally, several TL tip variants with bulky side chains (K1092W, K1093Y, K1093M) conferred GOF phenotypes (Fig 4B). Given the complexity and observation of both GOF/LOF phenotypes, we wished to further assess the functions of these residue-residue interactions.

Functional interactions among residues can be explored by the similarity between single substitution variants and the phenotypes of double mutants. We first sought evidence that variants in potential TL interaction partners could confer similar GOF or LOF phenotypes. In the simulation, K1092 switched interaction partners between two funnel helix residues D716 and E712 [18], and other charged residues were either observed or simulated to interact with S1091, K1092 or K1093 (Fig 5A). Therefore, we constructed a panel of mutants in the residues D716, E712, R1281, E1307, and D1309 for phenotypic analyses. Notably, we observed GOF phenotypes (Mn^S^ and MPA^S^) in E1307K but not E1307A, suggesting that E1307K gained an interfering interaction to destabilize the open TL state. Furthermore, we observed the Gal^R^ phenotype in D716A (Fig 5D, S8F Fig), consistent with LOF. D716K and E712A were lethal (Fig 5D, S8B and S8C Fig), and their defects were further explored by double mutant analyses (discussed below). Together, both GOF and LOF variants were observed in the TL tip proximal residues, consistent with roles in regulating TL dynamics.

To further dissect functional relationships, we phenotyped double mutants from potential interaction partners, and observed a number of genetic interactions (Fig 5D, S9 Fig). First, GOF and LOF mutants were mutually suppressive when combined, and most TL mutants from same biochemical class (GOF/GOF or LOF/LOF) showed additive effects (synthetically sick or lethal). The observed class-specific genetic interactions are similar to the previously reported intra-TL genetic interactions [37], consistent with alteration of TL function in TL tip proximal variants. Furthermore, K1092A/D single substitutions did not confer transcription-related phenotypes, but were able to suppress the E1307K GOF phenotypes. This observed epistasis suggested that loss of K1092 relieved a putative gain of interaction in E1307K (discussed above). Finally, E712A lethality was fully suppressed by K1092A, K1092D or K1093M, adding an additional instance of epistasis. A model to explain this complex genetic relationship is that loss of native E712-K1092 interaction re-directed K1092 towards an alternative interaction or strengthened an existing interaction with D716, causing lethality. Alteration of TL tip interaction potential through K1092/1093 substitutions relieves this allele-specific effect. Taken together, the observed allele-specific and epistatic interactions between TL tip and proximal residues suggest a highly complex genetic network of residues controlling TL dynamics, and illustrate how individual residues might constrain or allow diversification of the TL through evolution.

### Functional interplay of the TL and Bridge helix (BH) domains

The BH is a strikingly conserved structural domain of multi-subunit RNA polymerases spanning the wide central cleft between polymerase “jaws”, adjacent to the active site and proximal to the TL [1,69,70]. Although the BH is a straight helix in most published structures [1–6], some *Thermus thermophilus* RNAP structures revealed a bent BH conformation proposed to support translocation [69]. This BH bending mechanism was supported by a number of simulation studies but has never been directly tested [1,11,25,69,70]. In the archaeal *Mja* RNAP, proline substitutions at two hinge-proximal residues M808 and S824 (equivalent to *Sce* Rpb1 M818 and T834) resulted in GOF, suggesting kinking by the proline substitution results in increased translocation or catalysis [11,71]. Furthermore, *Mja* GOF TL and BH mutants were not additive when combined, suggesting mutual dependence on BH and TL functions [11].

**Fig 6:**
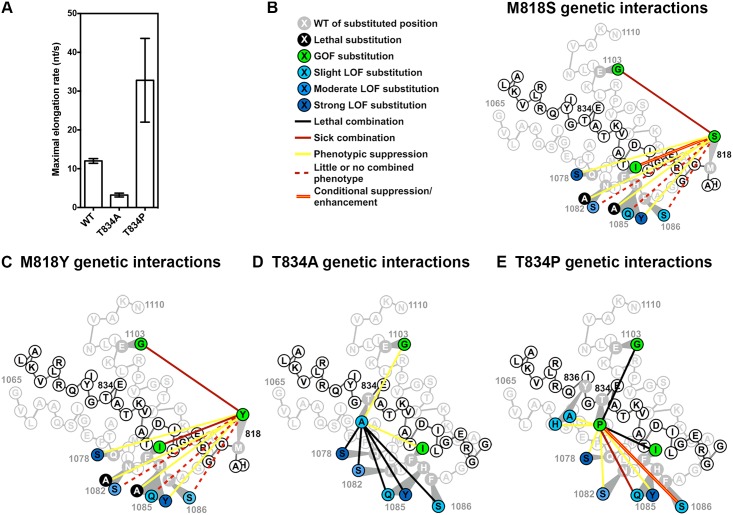
Functional interplay between the TL and Bridge Helix (BH). (A) Maximal *in vitro* elongation rates (nucleotides/second) of BH variants T834A and T834P. (B) Genetic interactions between BH M818S and TL substitutions. M818S suppressed (yellow lines) the strong LOF TL variants (dark blue) but not the slight and moderate LOF TL variants (light blue), and showed synthetic sickness (red lines) with the GOF TL variants (green). (C) Genetic interactions between BH M818Y and TL substitutions. Similar to M818S genetic interactions with TL variants (Fig 6B), M818Y suppressed (yellow lines) the strong LOF TL variants (dark blue) but not the slight and moderate LOF TL variants (light blue), and showed synthetic sickness (red lines) with GOF TL variants (green). (D) Genetic interactions between BH T834A and TL substitutions. T834A suppressed (yellow lines) the GOF TL variants and was synthetic lethal with all the tested LOF TL variants (blue). (E) Genetic interactions between BH T834P and TL or BH. Similar to M818 variants (Fig 6B, 6C), T834P suppressed strong and moderate LOF TL variants (dark blue) but was synthetic sick with weak LOF TL variants (light blue), while synthetically lethal with GOF TL variants (green). T834P was also suppressed (yellow line) by two LOF BH mutants Y836A/H.

To explore the functional consequence of BH kinking in *Sce* Pol II, we constructed and phenotyped BH mutants analogous to the characterized GOF and LOF variants in *Mja* RNAP. Notably, *Sce* T834 and other BH C-terminal hinge substitutions conferred *in vivo* phenotypes consistent with the altered transcriptional activities in *Mja* RNAP (S10F Fig), and we directly confirmed the altered activity of T834 variants *in vitro* (Fig 6A). In contrast, substitutions in M818, a predicted BH N-terminal hinge, showed defects deviating from expected conservation of function. M818P caused lethality, and could not be suppressed by any tested TL variants, precluding us from classifying it (S10A Fig). Furthermore, M818S and M818Y, although viable, did not confer any clear phenotypes (S10F Fig). Therefore, we further assessed the functional interplay between BH and TL by double mutant analyses, including BH variants (M818S/Y, T834A/P) and TL substitutions covering a range of altered transcriptional activities (Fig 6B–6E). Notably, the GOF BH variant T834P, along with M818S and M818Y, were mutually suppressive with biochemically strong LOF TL variants (Fig 6B, 6C and 6E), revealing both additive behavior between BH and TL for some combinations, and cryptic phenotypes for M818S/Y in others. The LOF BH variant T834A also suppressed GOF TL variants (Fig 6D). However, the additive interactions (exacerbation, synthetic lethality) we observed for GOF BH and TL double mutants were in contrast to the epistasis for *Mja* RNAP [11].

Multiple lines of evidence suggested additional, specific defects exist in BH mutants, beyond simple cooperation with the TL. First, M818P lethality could not be suppressed by any tested TL variants (S10A Fig), which cover a wide range of transcriptional activities. Second, suppression between BH and TL mutants of different biochemical classes (GOF/LOF) was partial and not as strong as the previously observed intra-TL suppression. Third, GOF M818S, M818Y and T834P variants appeared to exhibit activity-dependent genetic interactions with TL variants. BH GOF variants suppressed strong LOF TL variants Q1078S and H1085Y but failed to suppress, or even exacerbated slightly LOF TL variants H1085Q and F1086S (Fig 6B, 6C and 6E), consistent with conditional epistasis, where GOF activity of BH variants can suppress either specific TL variants or otherwise exert their effects in specific contexts. Finally, recent modeling studies predicted that the BH residue Y836 assists Pol II forward translocation [72] by interacting with the DNA:RNA hybrid. Y836A/H conferred Gal^R^ phenotypes, consistent with LOF and compromised translocation (S10F Fig). Notably, GOF T834P was suppressed by Y836A/H (Fig 6E, S12B Fig), consistent with T834P conferring a TL-independent fast translocation defect, suppressible by Y836A/H.

### Context dependence of TL function

**Fig 7:**
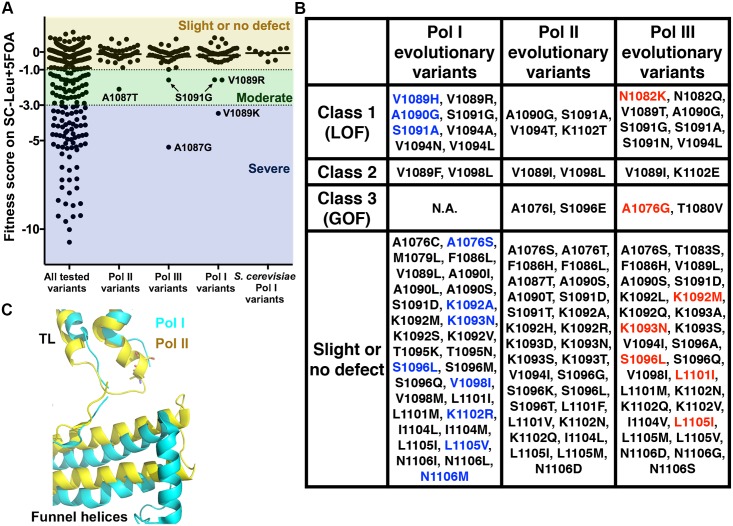
Phenotypic analyses of evolutionary variants suggest context-dependent functions for many TL residues. (A) General growth fitness defects of the TL single-substituted variants observed in the TL across Pol I, II, III evolution including 38 Pol II, 42 Pol I and 42 Pol III amino acid variants relative to *Sce* Pol II. (B) Evolutionary TL variants in three mutant classes from the TL phenotypic landscape (Fig 4A and 4B). Existing variants from *Sce* Pol I are colored in blue, and existing variants from *Sce* Pol III are colored in red. *Sce* Pol I has three substitutions (V1089H, A1090G and S1091A) that cause LOF in the Pol II context; *Sce* Pol III has one substitution (A1076G) classified as GOF and one substitution (N1082K) classified as LOF. (C) Difference in positioning of funnel helices (relative to TL) in Pol I and Pol II. Cartoon representation of TL/funnel helices from Pol I and Pol II are shown in cyan and yellow, respectively (PDB: 5C4J and 2VUM).

We previously observed that E1103G, a GOF allele in *Sce* Pol II, caused LOF in Pol I, highlighting divergent contributions of active site residues in different enzymatic contexts [42]. We also observed that the Pol I TL [42] and L1081M (this study) were functionally impaired in the Pol II context. We next sought to determine the functional compatibility of other evolutionary TL variants in the *Sce* Pol II context, using our fitness and phenotypic landscape (Fig 7). Most tested evolutionary TL variants did not confer fitness defects, with several exceptions (Fig 7A). Furthermore, some variants, although compatible for general growth, conferred transcription-related phenotypes and could be further classified by our phenotypic landscape (Fig 7B). These observations further suggest that the evolution of TL function is shaped by likely epistasis between the TL and proximal domains.

We next asked what substitutions might underlie the large difference in compatibility of the *Sce* Pol I TL (versus the *Sce* Pol III TL) within Pol II [42]. From our phenotypic landscape, although many individual *Sce* Pol I and Pol III TL substitutions appeared to be compatible, functionally impairing variants were identified (Fig 7B). The yeast Pol III TL contains Pol II GOF (A1076G) and LOF (N1082K) variants, both of which hypothetically could be mutually suppressive, resulting in close to WT activity in the Pol II context [42]. The Pol I TL contains three Pol II LOF substitutions (V1089H, A1090G and S1091A). The net incompatibility of Pol I TL is consistent with additive defects of the three LOF variations, given that most TL LOF combinations show additive effects [37]. Since three evolutionarily observed variants with LOF phenotypes were all localized in the TL tip, we examined the difference between Pol I and Pol II structures for the TL tip proximal domains [60,73]. The Pol I funnel helix appears to impose less constraint than the Pol II funnel helix (Fig 7C), suggesting that Pol I controls its TL with a distinct network of interactions. In all, our mutational data, together with the recent Pol I crystal structure, reveal enzyme-specific mechanisms to control a highly conserved domain at the heart of eukaryotic transcription.

## Discussion

The ability of the TL to fold into multiple conformations and the dynamic conversion between these states are critical for its functions. Previous studies from us and others demonstrate that TL function is delicately balanced, such that perturbations result in either increased or decreased catalytic activity and altered translocation dynamics. Distinct consequences for transcriptional activity manifest *in vivo* as what we term LOF and GOF phenotypes. In this study, we have advanced our genetic framework with which to dissect Pol II mechanisms. From our phenotypic landscape, we assessed the functional contributions of almost all TL residues to fitness in *S*. *cerevisiae* under multiple conditions. Our data indicate that both intra-TL interactions and TL interactions with nearby domains (*e*.*g*. BH and funnel helices) are critical for TL function. This conclusion is also supported by recent work on Rpb9 organizing the TL indirectly through an Rpb1 TL-adjacent α-helix 21 (one of the funnel helices, discussed below) [68], interactions between the TL and F-loop regions in bacteria [31], and predictions of TL-proximal variants as GOF from our previous pEMAP analysis [41] (validated in this study). Our system allows efficient analysis of a large number of variants to evaluate accumulating computational [18,24,25,74] and structural [4,5,27,60] predictions for interactions within the TL and from without.

The major function of the TL is to link substrate recognition to catalysis, while it is also proposed to gate translocation such that translocation probability is linked to phosphodiester bond formation. Critical to this recognition is that a substrate be positioned correctly by base-pairing to the DNA template, and that the 2’-OH allows NTPs to be selected over 2’-dNTPs by the TL residue Q1078 [4,9,28]. We have proposed that the Q1078-substrate interaction releases the adjacent M1079 from its intra-TL hydrophobic pocket to trigger TL closing [60]. In this study, we find a great number of variants within the pocket residues A1076, M1079, G1097, L1101 to cause GOF phenotypes, providing evidence that disruption of the hydrophobic pocket destabilizes the open, inactive TL state. Additionally, while the TL shows incredibly high evolutionary conservation for a number of residues, prior work indicated alteration of ultra-conserved residues (eg. E1103 in Pol II, E1224 in Pol I) in different RNA polymerases could have distinct effects, suggesting the importance of the evolved context within each enzyme [12,37,42]. Here, we evaluate many evolutionarily observed eukaryotic TL variants in the *Sce* Pol II system, and discover a number of functionally impaired TL variants. Our results highlight that TL proximal domains may impose constraint and also allow functional diversification in the molecular evolution of the highly conserved TL by epistatic interactions.

One example of a proximal region, the so-called “funnel helices” (Rpb1 α-20 and α-21) or “rim helices” in the bacterial RNAP literature, shows both evolutionary conservation and functional diversification. Funnel helices are both surface exposed and proximal to the TL [60,75]. Multiple pieces of evidence from three mutations in α-21 suggest roles for funnel helices in controlling TL function. One, the C4 allele of *Drosophila melanogaster*, corresponding to R726H in *Sce* Rpb1, confers a slow elongation rate in both Drosophila (*in* vitro) and human Pol II enzymes (in cells) [76,77]. The molecular mechanism of this allele is not currently known, but based on another α-21 substitution (G730D) identified in yeast, we would speculate C4 enzymes have altered TL dynamics. *rpb1-*G730D was identified in yeast twice, in independent genetic screens [78–80]. *rpb1-*G730D is catalytically slow [81], confers a severe growth defect but can be suppressed by a GOF mutant, *rpb9*Δ [68,78]. In fact, *rpb1-G730D* behaves as if it is incompatible with Rpb9 [68]. Recent work from the Peterson lab strongly supports a model where Rpb9 normally coordinates a loop of Rpb1 –the “anchor loop”–to appropriately interact with the TL [68]. When Rpb9 is removed, anchor loop-TL interactions are disrupted, and the open conformation of the TL is destabilized. In *rpb1-G730D*, structural perturbations are proposed to alter Rpb9-Rpb1 interactions such that they interfere with the TL, therefore *rpb1-G730D* is incompatible with Rpb9. Removal of Rpb9 or alteration of specific Rpb9 residues that organize the Rpb1 anchor loop relieve the incompatibility between *rpb1-G730D* and the TL. Third, we previously identified *rpb1-S713P*, a substitution just proximal to the anchor loop (between α-20 and α-21), as conferring gene expression, genetic interaction, and initiation phenotypes indistinguishable from GOF TL mutants [41]. Here we show that *rpb1-S713P* also confers increased biochemical activity, similar to both TL GOF alleles and anchor loop GOF alleles. We propose that *rpb1-S713P*, through constraints of the proline on structure, alters the anchor loop and therefore TL dynamics. It is conceivable, given that the secondary channel and funnel helices are accessible to factors, factor binding might also be communicated to the TL from distal sites. In addition to the three previously identified mutants, we utilized a new set of TL mutants to assess genetic interactions between the TL and the funnel helix α-21, and discover epistasis between K1092A/D (TL) and a lethal mutant E712A (funnel helix) along with multiple allele-specific genetic interactions (Fig 5D). We have suggested a more relaxed control mechanism in the Pol I compared to Pol II (Fig 7C). Taken together, funnel helices may serve as a regulatory hotspot for direct or allosteric control of the Pol II active site through the TL. While structurally conserved, evolutionary diversification of sequence may allow distinct interactions with the TL in different msRNAPs.

The characterization of the unexpectedly healthy H1085L variant clouds the issue of how H1085 functions in substrate selection and catalysis. H1085 interacts with the substrate NTP through salt bridge and hydrogen bond [4], and previous simulations with limiting H1085 variants predicted the hydrogen bonding to be critical for maintaining substrate interaction [74]. The discovery of H1085L argues that productive substrate interactions may be supported by entirely different chemistry, although we cannot rule out the possibility that H1085L redirects substrate interactions to an alternative residue. Furthermore, H1085 variants may have multiple defects in NAC, such as substrate selection [12], catalysis [12,61], intrinsic cleavage [61] and PPi release [20,21], and whether or not H1085 or analogous residues act as a general acid remains controversial in different RNAPs [4,7,9,61,82]. Function of H1085L in all of these steps remains to be determined, but the H1085L phenotype suggests that function of H1085 as a general acid may be entirely bypassed.

The established TL phenotypic landscape can be further explored to study intra- and inter-TL epistasis. First, whether individual TL residues work collaboratively or independently to ensure balanced TL dynamics and proper function is an open question. Some TL residues can be functionally overlapping and act at similar steps, or functionally discrete, acting at distinct steps. For example, combination of LOF mutations in Q1078, N1082 and a TL-proximal residue N479 resulted in non-additive genetic interaction, suggesting functionally overlapping roles for these residues. In contrast, combination of variants from Q1078 (or N1082) and H1085 resulted in exacerbation or synthetic lethality, suggesting independent functions [37]. Coupled with structures of partially folded TL states, these genetic studies support the functional distinction between NIR residues and a multi-step TL folding model for the promotion of catalysis [37]. Here, we have identified many more predicted GOF and LOF TL variants (Fig 4B), some of which are predicted to confer epistatic interactions (*e*.*g*. F1086 and V1089). We expect the phenotypic landscape of a multiply-substituted TL library to be extremely informative for understanding functional relationship between TL residues.

Second, the TL phenotypic landscape is an extremely sensitive readout for assessing active site re-arrangement. Transcription is under control by many factors, some of which may alter the Pol II active site conformations, though few studies directly address these possibilities. Initiation factors and Pol II TL mutants confer similar alterations in transcription start site selection, consistent with initiation factors functioning through the Pol II active site and altering the efficiency of Pol II catalysis during initiation [41,61,83]. Furthermore, TL may communicate with other Pol II sites, such as the RNA exit channel or clamp domain [36], or in direct competition with external factors, such as TFIIS [33]. Perturbations of this communication may alter TL dynamics and cause allele-specific genetic interactions (Figs 5 and 6). Specifically, an external perturbation by a relevant factor or Pol II TL distant domain may show epistasis or synergy only with specific TL alleles of a class (either LOF or GOF), whereas a non-interacting factor may not. Finally, similar perturbation of the TL phenotypic landscape by different factors would suggest functional similarity between them, thus clustering of phenotypic landscape changes upon different perturbations is expected to provide valuable insight.

The TL phenotypic landscape, along with our previous work [37], illustrates a strategy of utilizing *in vivo* genetic reporters or stress response profiles to distinguish a large number of mutants with distinct *in vivo* defects. As discussed above, the phenotypic landscape sheds light on functional contribution of TL residues to its dynamics, to the mechanism of catalysis and to the evolutionary constraints of the TL sequence and function. The phenotypic landscape strategy expands the current scope of existing deep mutational scanning studies [44–47], and can be generalized to study most, if not all, of the yeast proteins.

## Materials & Methods

### Yeast strains, media and plasmids

All yeast strains are derived from a *GAL2*^*+*^ derivative of S288C [84]. Genotypes of yeast strains are in S2 Table. Standard yeast media and the media for assessing established transcription-related phenotypes are as described previously [37]. For studies with 15 mM caffeine (Sigma), 150 mM hydroxyurea (Sigma), 5 mM and 15 mM MnCl_2_ (Sigma), 0.5 M NaCl (EMD), 3% formamide (JT Baker), 6% ethanol (KOPTEC), 0.07 μg/mL cycloheximide (Sigma), 10 mM HCl (EMD), 10 mM NaOH (EMD), 10 μg/mL benomyl (Sigma), each compound was added to the minimal SC-Leucine (SC-Leu) medium at the indicated concentration from concentrated stock solutions.

Detailed description of plasmids is in S2 Table, and complete sequences of plasmids are available upon request. For studies involving individual analyses of Pol II mutants, site-directed mutagenesis was performed via the Quickchange strategy from Stratagene. All mutagenized regions have been verified by sequencing before sub-cloning into pRS315-derived plasmids, as previously described [37].

### Genetic and biochemical analyses of individual Pol II mutants

Phenotypic analyses of individual Pol II mutants were performed by plasmid shuffling assays, with viable mutants further subjected to standard plate phenotyping. Each mutant in a pRS315-derived plasmid (*CEN LEU2*) was transformed into CKY283 (*rpb1*Δ::*CLONATMX*, pRP112 *RPB1 CEN URA3*). Transformants (Leu^+^) were patched on SC-Leu plates and subsequently replica plated to SC-Leu+5FOA (1mg/mL) to assay complementation ability upon loss of the *RPB1 CEN URA3* plasmid. Experimental details are as previously described [12,37]. Saturated cultures from single colonies of viable and shuffled Pol II mutants were subject to 10-fold serial dilution and spotting on indicated phenotyping media, as described in various previous reports [12,37].

Pol II enzymes were purified via a tandem-affinity tag (TAP) protocol derived from [85] with modifications described in [12]. Transcription elongation reactions were performed with Pol II elongation complexes assembled on a nucleic acid scaffold, in a procedure described in [12] with slight modifications in the amount of Pol II and nucleic acids as described in [60]. For each enzyme, elongation assays were performed with 25 μM, 125 μM, 500 μM and 750 μM NTPs (each of ATP, GTP, CTP, UTP), and maximal elongation rates were extracted exactly as previously described [12].

*ADH1* transcription start site selection was analyzed by primer extension. In brief, indicated strains were grown in YPD until mid-log phase (~1×10^7^ cells/mL), and diluted with YPD with 10mM MnCl_2_ or equal volume of H_2_O. Total RNA was extracted as described [86], and 30 μg of total RNA was subject to primer extension analysis, following a protocol derived from [87] with modifications described in [37].

### High-throughput phenotypic analyses of the TL variants library

The TL variant library was synthesized by Sloning Biotechnology (now MorphoSys) with well-characterized TL variants excluded (specified in Fig 1B) using a building block approach [48,49]. The TL variant library was transformed into CKY283 via a gap-repair strategy as previously described [41]. In brief, the amplified TL variant library with flanking sequence was transformed into CKY283 together with a linearized pRS315-derived plasmid (*CEN LEU2*) containing *rpb1* deleted for the TL (*TL*Δ) and linearized at the deletion junction, allowing *in vivo* homologous recombination. Homologous recombination produced a library of complete *rpb1* genes containing TL variants. The gap-repaired TL variants (Leu^+^) were titered and plated at 200–300 colonies per plate to reduce inter-colony growth competition, and Leu^+^ colonies were first replica-plated to SC-Leu+5FOA (1mg/mL), and subsequently to additional selective and control media. Three independent biological replicate screens were performed. In each replicate, we pooled 6000 to 12000 colonies. Each cell pool was subjected to genomic DNA extraction and TL amplification by emulsion PCR. Amplification of the TL region was performed using Micellula DNA Emulsion & Purification (ePCR) Kit (Chimerx) per manufacturer’s instructions. To minimize amplification bias, each sample was amplified in a 15-cycle ePCR reaction, purified and subject to additional 13–15 cycle scale-up ePCR reactions. The two-step ePCR amplification protocol ensured sufficient yield of DNA for NGS sequencing while minimizing perturbation of the allele distribution in the DNA pool. The amplified samples were subject to Illumina HiSeq 2500 sequencing, and on average over 2 million reads were obtained from each replicate of a sample, with high reproducibility and minimal perturbation of the mutant distribution within the TL variant library (S1D Fig).

Allele frequency was subsequently measured by deep sequencing of the TL amplicons. All the sequencing data (FASTQ format) for the reported analyses are deposited and available under the NCBI bioproject PRJNA340979. To identify the mutations that were present for each set of paired-end reads, a codon-based alignment algorithm was developed to align each paired-end read set in which the overlapping substrings from both flanking regions agreed perfectly to the WT sequence. The purpose of our approach was to identify real variants using an expected set of mutant codons used in the programmed library synthesis from sequencing errors. A dynamic programming algorithm was applied so that an exact match of three letters was assigned a positive score, a mismatch of at least one letter in a codon was assigned a negative score, and the insertion or deletion of either one, two or three letters was assigned a constant negative score. The allele frequency was subsequently calculated from the mapped reads, and the phenotypic score of each TL variant was calculated by allele frequency change (normalized to WT) under each condition, as below:

$$f=\text{log}\left[\frac{f^{mut,sele}}{f^{mut,unsele}}\right]-\text{log}\left[\frac{f^{wt,sele}}{f^{wt,unsele}}\right]$$

Mutants with less than 200 reads in the transformed pool (SC-Leu) and allele frequency changes assessed from less than 50 reads from both conditions were excluded from further analyses. Median values from three independent biological replicates were used for fitness and phenotype scoring. Fitness score cutoff for lethality was estimated based on fitness scores (on SC-Leu and 5FOA) of 163 known viable TL and 16 known lethal mutants. Hierarchical clustering for generating phenotypic landscape was performed by Gene Cluster 3.0 using centered correlation [88]. Figures displaying structural information were generated using Pymol (https://www.pymol.org/).

### Evolutionary analyses

Eukaryotic RNA polymerase large subunit sequences were obtained from BLAST using *Sce* Rpb1 (Pol II), *Sce* Rpa190 (Pol I), and *Sce* Rpo31 (Pol III) sequences as queries. Sequences were assigned to Pol I, II, or III based on highest similarity when compared to each of the three query sequences, with prokaryotic sequences further filtered out. Multiple sequence alignments (MSAs) were generated by first applying CD-HIT [89] to cluster sequences so that the identity between sequences in different clusters was less than 90%, then applying MUSCLE [90] to obtain an alignment that contains one representative sequence from each cluster. The TL conservation score was generated using Jalview 2.8 version 14.0 [91] and plotted as a heatmap using Gene-E (http://www.broadinstitute.org/cancer/software/GENE-E/index.html).

## Supporting Information

S1 Table Pol II mutants isolated in traditional screening of TL variant libraries and their phenotypes. (XLSX)

S2 Table Yeast strain genotypes and plasmid descriptions. (XLSX)

S1 Fig TL variant library composition and screening reproducibility. (A) Fraction of TL substitutions at each position of the TL (Rpb1 1076–1106). Allele frequencies were determined by deep sequencing of the TL variant library, and calculated by the number of reads from all the variants at a position divided by the total number of mapped reads.
(B) Fraction of TL substitutions for codons encoding specific amino acids. The allele frequency for each substitution was determined by deep sequencing of the TL variant library, calculated by the number of reads for variants substituted at a particular substitution divided by the total number of mapped reads.
(C) Distribution of allele frequencies for the detected TL single substitution variants.
(D) The TL library is robust to PCR amplification and yeast transformation. Pearson correlation coefficients calculated between different libraries are shown as a heatmap. TL library (Lib), PCR amplified TL library (Lib_PCR) and two yeast pools independently transformed with TL library (SC-Leu_screen1 and SC-Leu_screen2) were amplified and sequenced in triplicate (rep1, rep2 and rep3), and pairwise Pearson correlation analyses were performed between different sequencing libraries.
(TIF)

S2 Fig Screening for allele-specific stress conditions by standard plate phenotyping of 50 isolated TL variants. 10-fold serial dilutions of saturated cultures of the 50 TL variants were plated on the indicated conditions, including 15 mM caffeine, 150 mM hydroxyurea, 5 mM Mn^2+^, 15 mM Mn^2+^ and 0.5 M NaCl.
(TIF)

S3 Fig Screening for additional allele-specific stress conditions for 50 isolated TL variants. 10-fold serial dilutions of saturated cultures of the 50 TL variants were plated on the indicated conditions, including 3% formamide, 6% ethanol, 0.07 μg/mL cycloheximide, 10 mM HCl, 10 mM NaOH and 10 μg/mL benomyl.
(TIF)

S4 Fig Transcription-related phenotypes of 50 isolated TL variants. Gal^R^, MPA^S^ and Spt^-^ phenotypes of the 50 TL variants were assessed as a control for the high-throughput phenotyping.
(TIF)

S5 Fig Structures of different TL states allow prediction of functionally important residue-residue interactions. (A) A1087-G1088 linker is highly spatially constrained. The closed TL (magenta) is shown in cartoon (A1087, G1088 in sticks), and TL-proximal domains are shown in surface representation. Rpb2 domains are colored in grey; Bridge Helix (Rpb1 800–860) in cyan; Funnel helix α-21 (Rpb1 700–750) in green.
(B) Change of F1086-V1089 interactions in different TL states. V1089 forms a backbone-backbone hydrogen bond with F1086 in the open TL (orange, PDB: 5C4X), but the side chain flips towards the F1086 for a hydrophobic interaction when the TL is in a less open state (yellow, PDB: 5C4J).
(C) V1094-K830 interaction in the closed TL state. The charged K830 side chain appears to be neutralized by D826 through a salt bridge interaction, and the neutralized K830 side chain interacts with the V1094 side chain.
(D) Observed hydrophobic pocket in the open TL surrounding M1079 (PDB: 5C4J). TL (yellow) and the proximal domains (cyan) are shown in the cartoon representation with the M1079-proximal hydrophobic residues shown in spheres. M1079 is highlighted in red.
(E) Transcription-related phenotypes of G1097 variants.
(TIF)

S6 Fig Phenotypic scores of TL variants under screened conditions. Phenotypic scores of TL single substituted variants under indicated conditions are shown in separate heatmaps. Unavailable data points are shown as filled grey squares. WT residues at indicated positions are outlined in black boxes. Predicted lethal mutants are in filled black squares.
(TIF)

S7 Fig Quantitation of maximal elongation rates for Pol II WT and mutant enzymes. (A-F) Determination of elongation rates at different NTP concentrations for indicated enzymes. Fraction of run-off transcripts by the total (Fraction elongated) was quantified and plotted versus reaction time for indicated Pol II mutants. Lines of different colors indicate the different concentrations of NTPs used. At least three experimental replicates were performed and each replicate was separately curve fitted with non-linear regression (GraphPad Prism 6.0h).
(G) Determination of maximal elongation rates for indicated enzymes. Elongation rates (determined from S7A-F Fig) were plotted versus NTP concentrations and curve fitted with non-linear regression (GraphPad Prism 6.0h).
(TIF)

S8 Fig Construction and transcription-related phenotypes of the TL tip and nearby charged residue variants. (A) *x*-*y* plot showing the lack of correlation between helical propensity change and phenotypic score on MPA, a good indicator of altered transcription activity. 120 variants from the TL tip region (top panel) and 104 variants from the same region but excluding V1094 mutants (bottom panel) are shown, with linear regression fit of the data shown in (some color).
(B-E) Complementation abilities of TL tip (S1091, K1092, K1093) variants, tip proximal D716 (B), E712 (C), E1307 (D), R1281 (E) variants and the corresponding double mutants were determined by plasmid shuffling assays.
(F) Transcription-related phenotypes of TL tip and the TL-proximal charged residue variants. S1091C, K1093M and E1307K confer MPA^S^ phenotypes, and K1093M additionally confers an Spt^-^ phenotype, while others alone don’t confer any strong transcription-related phenotypes.
(TIF)

S9 Fig Genetic interactions of the TL tip and nearby charged residue variants on transcription-related phenotypes. Genetic interactions between tip variants and nearby charged residues D716 (A), E712 (B), R1281 (C) and E1307 (D, E) variants detected by alterations in transcription-related phenotypes.
(TIF)

S10 Fig Construction and transcription-related phenotypes of TL and BH variants. (A-E) Complementation ability of the indicated TL variants, BH single variants M818P (A), M818S (B), M818Y (C), T834A (D), T834P (E) and the corresponding double/triple mutants were determined by plasmid shuffling assays.
(F) Transcription-linked phenotypes of BH single-substituted mutants. M818S and M818Y are substitutions in a predicted BH N-terminal hinge; others are substitutions in predicted BH C-terminal hinge positions or additional C-terminal substitutions.
(TIF)

S11 Fig Genetic interactions of BH M818 and TL mutants for transcription-related phenotypes. Genetic interactions between TL variants and the BH variants M818S (A), M818Y (B) were assessed by standard plate phenotyping of transcription-related phenotypes.
(TIF)

S12 Fig Genetic interactions of BH T834 and TL mutants on transcription-related phenotypes. Genetic interactions between TL variants and the BH variants T834A (A), T834P (B) were assessed by standard plate phenotyping of transcription-related phenotypes. Additional genetic interactions between T834P (GOF) and two LOF BH mutants (Y836A) and Y836H are included in (B).
(TIF)

## References

[1] Gnatt AL, Cramer P, Fu J, Bushnell DA, Kornberg RD (2001) Structural basis of transcription: an RNA polymerase II elongation complex at 3.3 A resolution. Science 292: 1876–1882. 10.1126/science.1059495 11313499

[2] Cramer P, Bushnell DA, Kornberg RD (2001) Structural basis of transcription: RNA polymerase II at 2.8 angstrom resolution. Science 292: 1863–1876. 10.1126/science.1059493 11313498

[3] Westover KD, Bushnell DA, Kornberg RD (2004) Structural basis of transcription: nucleotide selection by rotation in the RNA polymerase II active center. Cell 119: 481–489. 10.1016/j.cell.2004.10.016 15537538

[4] Wang D, Bushnell DA, Westover KD, Kaplan CD, Kornberg RD (2006) Structural basis of transcription: role of the trigger loop in substrate specificity and catalysis. Cell 127: 941–954. 10.1016/j.cell.2006.11.023 17129781 PMC1876690

[5] Liu X, Bushnell DA, Kornberg RD (2013) RNA polymerase II transcription: structure and mechanism. Biochim Biophys Acta 1829: 2–8. 10.1016/j.bbagrm.2012.09.003 23000482 PMC4244541

[6] Kaplan CD (2013) Basic mechanisms of RNA polymerase II activity and alteration of gene expression in Saccharomyces cerevisiae. Biochim Biophys Acta 1829: 39–54. 10.1016/j.bbagrm.2012.09.007 23022618 PMC4026157

[7] Zhang J, Palangat M, Landick R (2010) Role of the RNA polymerase trigger loop in catalysis and pausing. Nat Struct Mol Biol 17: 99–104. 10.1038/nsmb.1732 19966797 PMC2904963

[8] Windgassen TA, Mooney RA, Nayak D, Palangat M, Zhang J, et al (2014) Trigger-helix folding pathway and SI3 mediate catalysis and hairpin-stabilized pausing by Escherichia coli RNA polymerase. Nucleic Acids Res 42: 12707–12721. 10.1093/nar/gku997 25336618 PMC4227799

[9] Yuzenkova Y, Bochkareva A, Tadigotla VR, Roghanian M, Zorov S, et al (2010) Stepwise mechanism for transcription fidelity. BMC Biol 8: 54 10.1186/1741-7007-8-54 20459653 PMC2874521

[10] Fouqueau T, Zeller ME, Cheung AC, Cramer P, Thomm M (2013) The RNA polymerase trigger loop functions in all three phases of the transcription cycle. Nucleic Acids Research 41: 7048–7059. 10.1093/nar/gkt433 23737452 PMC3737540

[11] Tan L, Wiesler S, Trzaska D, Carney HC, Weinzierl RO (2008) Bridge helix and trigger loop perturbations generate superactive RNA polymerases. J Biol 7: 40 10.1186/jbiol98 19055851 PMC2776397

[12] Kaplan CD, Larsson KM, Kornberg RD (2008) The RNA polymerase II trigger loop functions in substrate selection and is directly targeted by alpha-amanitin. Mol Cell 30: 547–556. 10.1016/j.molcel.2008.04.023 18538653 PMC2475549

[13] Kireeva ML, Nedialkov YA, Cremona GH, Purtov YA, Lubkowska L, et al (2008) Transient reversal of RNA polymerase II active site closing controls fidelity of transcription elongation. Mol Cell 30: 557–566. 10.1016/j.molcel.2008.04.017 18538654 PMC7243879

[14] Fong N, Kim H, Zhou Y, Ji X, Qiu J, et al (2014) Pre-mRNA splicing is facilitated by an optimal RNA polymerase II elongation rate. Genes Dev 28: 2663–2676. 10.1101/gad.252106.114 25452276 PMC4248296

[15] Malinen AM, Turtola M, Parthiban M, Vainonen L, Johnson MS, et al (2012) Active site opening and closure control translocation of multisubunit RNA polymerase. Nucleic Acids Res 40: 7442–7451. 10.1093/nar/gks383 22570421 PMC3424550

[16] Xu L, Butler KV, Chong J, Wengel J, Kool ET, et al (2014) Dissecting the chemical interactions and substrate structural signatures governing RNA polymerase II trigger loop closure by synthetic nucleic acid analogues. Nucleic Acids Res 42: 5863–5870. 10.1093/nar/gku238 24692664 PMC4027217

[17] Nayak D, Voss M, Windgassen T, Mooney RA, Landick R (2013) Cys-pair reporters detect a constrained trigger loop in a paused RNA polymerase. Mol Cell 50: 882–893. 10.1016/j.molcel.2013.05.015 23769674 PMC4037917

[18] Wang B, Predeus AV, Burton ZF, Feig M (2013) Energetic and structural details of the trigger-loop closing transition in RNA polymerase II. Biophys J 105: 767–775. 10.1016/j.bpj.2013.05.060 23931324 PMC3736665

[19] Vassylyev DG, Vassylyeva MN, Zhang J, Palangat M, Artsimovitch I, et al (2007) Structural basis for substrate loading in bacterial RNA polymerase. Nature 448: 163–168. 10.1038/nature05931 17581591

[20] Da LT, Wang D, Huang X (2012) Dynamics of pyrophosphate ion release and its coupled trigger loop motion from closed to open state in RNA polymerase II. J Am Chem Soc 134: 2399–2406. 10.1021/ja210656k 22206270 PMC3273452

[21] Liu B, Zuo Y, Steitz TA (2016) Structures of E. coli sigmaS-transcription initiation complexes provide new insights into polymerase mechanism. Proc Natl Acad Sci U S A 113: 4051–4056. 10.1073/pnas.1520555113 27035955 PMC4839411

[22] Toulokhonov I, Zhang J, Palangat M, Landick R (2007) A central role of the RNA polymerase trigger loop in active-site rearrangement during transcriptional pausing. Mol Cell 27: 406–419. 10.1016/j.molcel.2007.06.008 17679091

[23] Larson MH, Zhou J, Kaplan CD, Palangat M, Kornberg RD, et al (2012) Trigger loop dynamics mediate the balance between the transcriptional fidelity and speed of RNA polymerase II. Proceedings of the National Academy of Sciences of the United States of America 109: 6555–6560. 10.1073/pnas.1200939109 22493230 PMC3340090

[24] Seibold SA, Singh BN, Zhang C, Kireeva M, Domecq C, et al (2010) Conformational coupling, bridge helix dynamics and active site dehydration in catalysis by RNA polymerase. Biochim Biophys Acta 1799: 575–587. 10.1016/j.bbagrm.2010.05.002 20478425 PMC2922424

[25] Silva DA, Weiss DR, Pardo Avila F, Da LT, Levitt M, et al (2014) Millisecond dynamics of RNA polymerase II translocation at atomic resolution. Proc Natl Acad Sci U S A 111: 7665–7670. 10.1073/pnas.1315751111 24753580 PMC4040580

[26] Weixlbaumer A, Leon K, Landick R, Darst SA (2013) Structural basis of transcriptional pausing in bacteria. Cell 152: 431–441. 10.1016/j.cell.2012.12.020 23374340 PMC3564060

[27] Wang D, Bushnell DA, Huang X, Westover KD, Levitt M, et al (2009) Structural basis of transcription: backtracked RNA polymerase II at 3.4 angstrom resolution. Science 324: 1203–1206. 10.1126/science.1168729 19478184 PMC2718261

[28] Cheung AC, Cramer P (2011) Structural basis of RNA polymerase II backtracking, arrest and reactivation. Nature 471: 249–253. 10.1038/nature09785 21346759

[29] Yuzenkova Y, Zenkin N (2010) Central role of the RNA polymerase trigger loop in intrinsic RNA hydrolysis. Proc Natl Acad Sci U S A 107: 10878–10883. 10.1073/pnas.0914424107 20534498 PMC2890756

[30] Sosunova E, Sosunov V, Epshtein V, Nikiforov V, Mustaev A (2013) Control of transcriptional fidelity by active center tuning as derived from RNA polymerase endonuclease reaction. J Biol Chem 288: 6688–6703. 10.1074/jbc.M112.424002 23283976 PMC5396497

[31] Miropolskaya N, Esyunina D, Klimasauskas S, Nikiforov V, Artsimovitch I, et al (2014) Interplay between the trigger loop and the F loop during RNA polymerase catalysis. Nucleic Acids Res 42: 544–552. 10.1093/nar/gkt877 24089145 PMC3874190

[32] Esyunina D, Turtola M, Pupov D, Bass I, Klimasauskas S, et al (2016) Lineage-specific variations in the trigger loop modulate RNA proofreading by bacterial RNA polymerases. Nucleic Acids Res 44: 1298–1308. 10.1093/nar/gkv1521 26733581 PMC4756841

[33] Kettenberger H, Armache KJ, Cramer P (2004) Complete RNA polymerase II elongation complex structure and its interactions with NTP and TFIIS. Mol Cell 16: 955–965. 10.1016/j.molcel.2004.11.040 15610738

[34] Lennon CW, Ross W, Martin-Tumasz S, Toulokhonov I, Vrentas CE, et al (2012) Direct interactions between the coiled-coil tip of DksA and the trigger loop of RNA polymerase mediate transcriptional regulation. Genes Dev 26: 2634–2646. 10.1101/gad.204693.112 23207918 PMC3521624

[35] Sekine S, Murayama Y, Svetlov V, Nudler E, Yokoyama S (2015) The ratcheted and ratchetable structural states of RNA polymerase underlie multiple transcriptional functions. Mol Cell 57: 408–421. 10.1016/j.molcel.2014.12.014 25601758

[36] Hein PP, Kolb KE, Windgassen T, Bellecourt MJ, Darst SA, et al (2014) RNA polymerase pausing and nascent-RNA structure formation are linked through clamp-domain movement. Nature Structural & Molecular Biology 21: 794–802. 10.1038/nsmb.2867 PMC4156911 25108353

[37] Kaplan CD, Jin H, Zhang IL, Belyanin A (2012) Dissection of Pol II trigger loop function and Pol II activity-dependent control of start site selection in vivo. PLoS Genet 8: e1002627 10.1371/journal.pgen.1002627 22511879 PMC3325174

[38] Malagon F, Kireeva ML, Shafer BK, Lubkowska L, Kashlev M, et al (2006) Mutations in the Saccharomyces cerevisiae RPB1 gene conferring hypersensitivity to 6-azauracil. Genetics 172: 2201–2209. 10.1534/genetics.105.052415 16510790 PMC1456368

[39] Irvin JD, Kireeva ML, Gotte DR, Shafer BK, Huang I, et al (2014) A genetic assay for transcription errors reveals multilayer control of RNA polymerase II fidelity. PLoS Genet 10: e1004532 10.1371/journal.pgen.1004532 25232834 PMC4168980

[40] Dangkulwanich M, Ishibashi T, Liu S, Kireeva ML, Lubkowska L, et al (2013) Complete dissection of transcription elongation reveals slow translocation of RNA polymerase II in a linear ratchet mechanism. Elife 2: e00971 10.7554/eLife.00971 24066225 PMC3778554

[41] Braberg H, Jin H, Moehle EA, Chan YA, Wang S, et al (2013) From structure to systems: high-resolution, quantitative genetic analysis of RNA polymerase II. Cell 154: 775–788. 10.1016/j.cell.2013.07.033 23932120 PMC3932829

[42] Viktorovskaya OV, Engel KL, French SL, Cui P, Vandeventer PJ, et al (2013) Divergent contributions of conserved active site residues to transcription by eukaryotic RNA polymerases I and II. Cell Rep 4: 974–984. 10.1016/j.celrep.2013.07.044 23994471 PMC3801175

[43] Mejia YX, Nudler E, Bustamante C (2015) Trigger loop folding determines transcription rate of Escherichia coli's RNA polymerase. Proc Natl Acad Sci U S A 112: 743–748. 10.1073/pnas.1421067112 25552559 PMC4311812

[44] Fowler DM, Fields S (2014) Deep mutational scanning: a new style of protein science. Nat Methods 11: 801–807. 10.1038/nmeth.3027 25075907 PMC4410700

[45] McLaughlin RN Jr., Poelwijk FJ, Raman A, Gosal WS, Ranganathan R (2012) The spatial architecture of protein function and adaptation. Nature 491: 138–142. 10.1038/nature11500 23041932 PMC3991786

[46] Araya CL, Fowler DM, Chen W, Muniez I, Kelly JW, et al (2012) A fundamental protein property, thermodynamic stability, revealed solely from large-scale measurements of protein function. Proc Natl Acad Sci U S A 109: 16858–16863. 10.1073/pnas.1209751109 23035249 PMC3479514

[47] Kim I, Miller CR, Young DL, Fields S (2013) High-throughput analysis of in vivo protein stability. Mol Cell Proteomics 12: 3370–3378. 10.1074/mcp.O113.031708 23897579 PMC3820947

[48] Zhai W, Glanville J, Fuhrmann M, Mei L, Ni I, et al (2011) Synthetic antibodies designed on natural sequence landscapes. J Mol Biol 412: 55–71. 10.1016/j.jmb.2011.07.018 21787786

[49] Van den Brulle J, Fischer M, Langmann T, Horn G, Waldmann T, et al (2008) A novel solid phase technology for high-throughput gene synthesis. Biotechniques 45: 340–343. 10.2144/000112953 18778261

[50] Tewhey R, Warner JB, Nakano M, Libby B, Medkova M, et al (2009) Microdroplet-based PCR enrichment for large-scale targeted sequencing. Nat Biotechnol 27: 1025–1031. 10.1038/nbt.1583 19881494 PMC2779736

[51] Williams R, Peisajovich SG, Miller OJ, Magdassi S, Tawfik DS, et al (2006) Amplification of complex gene libraries by emulsion PCR. Nat Methods 3: 545–550. 10.1038/nmeth896 16791213

[52] Simchen G, Winston F, Styles CA, Fink GR (1984) Ty-mediated gene expression of the LYS2 and HIS4 genes of Saccharomyces cerevisiae is controlled by the same SPT genes. Proc Natl Acad Sci U S A 81: 2431–2434. 6326126 10.1073/pnas.81.8.2431 PMC345074

[53] Cui P, Jin H, Vutukuru MR, Kaplan CD (2016) Relationships Between RNA Polymerase II Activity and Spt Elongation Factors to Spt- Phenotype and Growth in Saccharomyces cerevisiae. G3 (Bethesda). 10.1534/g3.116.030346 PMC4978902 27261007

[54] Greger IH, Proudfoot NJ (1998) Poly(A) signals control both transcriptional termination and initiation between the tandem GAL10 and GAL7 genes of Saccharomyces cerevisiae. EMBO J 17: 4771–4779. 10.1093/emboj/17.16.4771 9707436 PMC1170806

[55] Kaplan CD, Holland MJ, Winston F (2005) Interaction between transcription elongation factors and mRNA 3'-end formation at the Saccharomyces cerevisiae GAL10-GAL7 locus. J Biol Chem 280: 913–922. 10.1074/jbc.M411108200 15531585

[56] Malik I, Qiu C, Snavely T, Kaplan CD (2016) Effects of Pol II catalytic mutants on in vivo elongation rate, processivity, gene expression, mRNA decay and response to nucleotide depletion. bioRxiv.

[57] Jenks MH, O'Rourke TW, Reines D (2008) Properties of an intergenic terminator and start site switch that regulate IMD2 transcription in yeast. Mol Cell Biol 28: 3883–3893. 10.1128/MCB.00380-08 18426909 PMC2423123

[58] Kuehner JN, Brow DA (2008) Regulation of a eukaryotic gene by GTP-dependent start site selection and transcription attenuation. Mol Cell 31: 201–211. 10.1016/j.molcel.2008.05.018 18657503

[59] Hyle JW, Shaw RJ, Reines D (2003) Functional distinctions between IMP dehydrogenase genes in providing mycophenolate resistance and guanine prototrophy to yeast. J Biol Chem 278: 28470–28478. 10.1074/jbc.M303736200 12746440 PMC3367515

[60] Barnes CO, Calero M, Malik I, Graham BW, Spahr H, et al (2015) Crystal Structure of a Transcribing RNA Polymerase II Complex Reveals a Complete Transcription Bubble. Mol Cell 59: 258–269. 10.1016/j.molcel.2015.06.034 26186291 PMC4643057

[61] Cabart P, Jin H, Li L, Kaplan CD (2014) Activation and reactivation of the RNA polymerase II trigger loop for intrinsic RNA cleavage and catalysis. Transcription 5: e28869 10.4161/trns.28869 25764335 PMC4574878

[62] Niyogi SK, Feldman RP, Hoffman DJ (1981) Selective effects of metal ions on RNA synthesis rates. Toxicology 22: 9–21. 6175051 10.1016/0300-483x(81)90003-2

[63] Niyogi SK, Feldman RP (1981) Effect of several metal ions on misincorporation during transcription. Nucleic Acids Res 9: 2615–2627. 7024904 10.1093/nar/9.11.2615 PMC326876

[64] Dyson HJ, Wright PE, Scheraga HA (2006) The role of hydrophobic interactions in initiation and propagation of protein folding. Proc Natl Acad Sci U S A 103: 13057–13061. 10.1073/pnas.0605504103 16916929 PMC1559752

[65] Cheung AC, Sainsbury S, Cramer P (2011) Structural basis of initial RNA polymerase II transcription. EMBO J 30: 4755–4763. 10.1038/emboj.2011.396 22056778 PMC3243610

[66] Rudolph HK, Antebi A, Fink GR, Buckley CM, Dorman TE, et al (1989) The yeast secretory pathway is perturbed by mutations in PMR1, a member of a Ca2+ ATPase family. Cell 58: 133–145. 2526682 10.1016/0092-8674(89)90410-8

[67] Mandal D, Woolf TB, Rao R (2000) Manganese selectivity of pmr1, the yeast secretory pathway ion pump, is defined by residue gln783 in transmembrane segment 6. Residue Asp778 is essential for cation transport. J Biol Chem 275: 23933–23938. 10.1074/jbc.M002619200 10801856

[68] Kaster BC, Knippa KC, Kaplan CD, Peterson DO (2016) RNA Polymerase II Trigger Loop Mobility: Indirect Effects of Rpb9. J Biol Chem. 10.1074/jbc.M116.714394 PMC4938204 27226557

[69] Vassylyev DG, Sekine S, Laptenko O, Lee J, Vassylyeva MN, et al (2002) Crystal structure of a bacterial RNA polymerase holoenzyme at 2.6 A resolution. Nature 417: 712–719. 10.1038/nature752 12000971

[70] Hirata A, Klein BJ, Murakami KS (2008) The X-ray crystal structure of RNA polymerase from Archaea. Nature 451: 851–854. 10.1038/nature06530 18235446 PMC2805805

[71] Weinzierl RO (2010) The nucleotide addition cycle of RNA polymerase is controlled by two molecular hinges in the Bridge Helix domain. BMC Biol 8: 134 10.1186/1741-7007-8-134 21034443 PMC2988716

[72] Da LT, Pardo-Avila F, Xu L, Silva DA, Zhang L, et al (2016) Bridge helix bending promotes RNA polymerase II backtracking through a critical and conserved threonine residue. Nat Commun 7: 11244 10.1038/ncomms11244 27091704 PMC4838855

[73] Engel C, Sainsbury S, Cheung AC, Kostrewa D, Cramer P (2013) RNA polymerase I structure and transcription regulation. Nature 502: 650–655. 10.1038/nature12712 24153182

[74] Huang X, Wang D, Weiss DR, Bushnell DA, Kornberg RD, et al (2010) RNA polymerase II trigger loop residues stabilize and position the incoming nucleotide triphosphate in transcription. Proc Natl Acad Sci U S A 107: 15745–15750. 10.1073/pnas.1009898107 20798057 PMC2936645

[75] Opalka N, Chlenov M, Chacon P, Rice WJ, Wriggers W, et al (2003) Structure and function of the transcription elongation factor GreB bound to bacterial RNA polymerase. Cell 114: 335–345. 12914698 10.1016/s0092-8674(03)00600-7

[76] de la Mata M, Alonso CR, Kadener S, Fededa JP, Blaustein M, et al (2003) A slow RNA polymerase II affects alternative splicing in vivo. Mol Cell 12: 525–532. 14536091 10.1016/j.molcel.2003.08.001

[77] Coulter DE, Greenleaf AL (1985) A mutation in the largest subunit of RNA polymerase II alters RNA chain elongation in vitro. J Biol Chem 260: 13190–13198. 2414275

[78] Koyama H, Ueda T, Ito T, Sekimizu K (2010) Novel RNA polymerase II mutation suppresses transcriptional fidelity and oxidative stress sensitivity in rpb9Delta yeast. Genes Cells 15: 151–159. 10.1111/j.1365-2443.2009.01372.x 20088966

[79] Archambault J, Jansma DB, Kawasoe JH, Arndt KT, Greenblatt J, et al (1998) Stimulation of transcription by mutations affecting conserved regions of RNA polymerase II. J Bacteriol 180: 2590–2598. 9573141 10.1128/jb.180.10.2590-2598.1998 PMC107208

[80] Arndt KT, Styles CA, Fink GR (1989) A suppressor of a HIS4 transcriptional defect encodes a protein with homology to the catalytic subunit of protein phosphatases. Cell 56: 527–537. 2537149 10.1016/0092-8674(89)90576-x

[81] Walmacq C, Cheung AC, Kireeva ML, Lubkowska L, Ye C, et al (2012) Mechanism of translesion transcription by RNA polymerase II and its role in cellular resistance to DNA damage. Mol Cell 46: 18–29. 10.1016/j.molcel.2012.02.006 22405652 PMC3329276

[82] Castro C, Smidansky ED, Arnold JJ, Maksimchuk KR, Moustafa I, et al (2009) Nucleic acid polymerases use a general acid for nucleotidyl transfer. Nat Struct Mol Biol 16: 212–218. 10.1038/nsmb.1540 19151724 PMC2728625

[83] Jin H, Kaplan CD (2014) Relationships of RNA polymerase II genetic interactors to transcription start site usage defects and growth in Saccharomyces cerevisiae. G3 (Bethesda) 5: 21–33. 25380729 10.1534/g3.114.015180 PMC4291466

[84] Winston F, Dollard C, Ricupero-Hovasse SL (1995) Construction of a set of convenient Saccharomyces cerevisiae strains that are isogenic to S288C. Yeast 11: 53–55. 10.1002/yea.320110107 7762301

[85] Puig O, Caspary F, Rigaut G, Rutz B, Bouveret E, et al (2001) The tandem affinity purification (TAP) method: a general procedure of protein complex purification. Methods 24: 218–229. 10.1006/meth.2001.1183 11403571

[86] Schmitt ME, Brown TA, Trumpower BL (1990) A rapid and simple method for preparation of RNA from Saccharomyces cerevisiae. Nucleic Acids Res 18: 3091–3092. 2190191 10.1093/nar/18.10.3091 PMC330876

[87] Ranish JA, Hahn S (1991) The yeast general transcription factor TFIIA is composed of two polypeptide subunits. J Biol Chem 266: 19320–19327. 1918049

[88] de Hoon MJ, Imoto S, Nolan J, Miyano S (2004) Open source clustering software. Bioinformatics 20: 1453–1454. 10.1093/bioinformatics/bth078 14871861

[89] Li W, Jaroszewski L, Godzik A (2002) Tolerating some redundancy significantly speeds up clustering of large protein databases. Bioinformatics 18: 77–82. 11836214 10.1093/bioinformatics/18.1.77

[90] Edgar RC (2004) MUSCLE: multiple sequence alignment with high accuracy and high throughput. Nucleic Acids Res 32: 1792–1797. 10.1093/nar/gkh340 15034147 PMC390337

[91] Waterhouse AM, Procter JB, Martin DM, Clamp M, Barton GJ (2009) Jalview Version 2—a multiple sequence alignment editor and analysis workbench. Bioinformatics 25: 1189–1191. 10.1093/bioinformatics/btp033 19151095 PMC2672624

