# Correction: High-Resolution Phenotypic Landscape of the RNA Polymerase II Trigger Loop

**DOI:** 10.1371/journal.pgen.1007158

**Published:** 2018-01-03

**Authors:** Chenxi Qiu, Olivia C. Erinne, Jui M. Dave, Ping Cui, Huiyan Jin, Nandhini Muthukrishnan, Leung K. Tang, Sabareesh Ganesh Babu, Kenny C. Lam, Paul J. Vandeventer, Ralf Strohner, Jan Van den Brulle, Sing-Hoi Sze, Craig D. Kaplan

This Research Article reports that lethality was observed for *rpb1* E712A and D716K. While rephenotyping mutants from this study, the authors found that a subclone with a frameshift was used in the construction of the authors’ *rpb1* D716K mutant and six D716K derived double mutants. In addition, they did not observe lethality of *rpb1* E712A. The authors are issuing a correction to rectify this mistake.

The phenotypes of the newly constructed and verified mutants are: *rpb1* D716K and *rpb1* E712A are viable (corrected in Figs 5D, S8B and S8C) and do not confer transcription-related plate phenotypes. All the reconstructed D716K containing double mutants (D716K/S1091A, D716K/S1091E, D716K/S1091C, D716K/K1092A, D716K/K1092D, D716K/K1093M) are also viable (previously reported as lethal in this publication). The phenotypes are as follows: D716K exacerbates the slight LOF phenotypes of S1091A and S1091E but suppresses the GOF phenotypes of S1091C and K1093M, consistent with D716K being LOF and the observed allele specific genetic interactions in the region.

The authors have verified that phenotypes of the following mutants are reported correctly in this publication: All the E712A containing double mutants (E712A/S1091A, E712A/S1091E, E712A/S1091C, E712A/K1092A, E712A/K1092D, E712A/K1093M) and the single substitution mutants T834P, S1091A, S1091E, S1091C, K1092A, K1092D, K1093M.

## Amendments to Fig 5, S8 Fig, S9 Fig and S2 Table

Panel D in Fig 5, and panels B and C in S8 Fig have been corrected to account for these changes. Panel F of S8 Fig and panels A and B of S9 Fig have been updated to include the observed phenotypes of the viable E712A, D716K and D716K-containing double mutants. S2 Table has also been updated to account for the changes. Please view the corrected Fig 5 and the corrected and updated S8 Fig, S9 Fig and S2 Table, along with the corresponding legends below.

**Fig 5:**
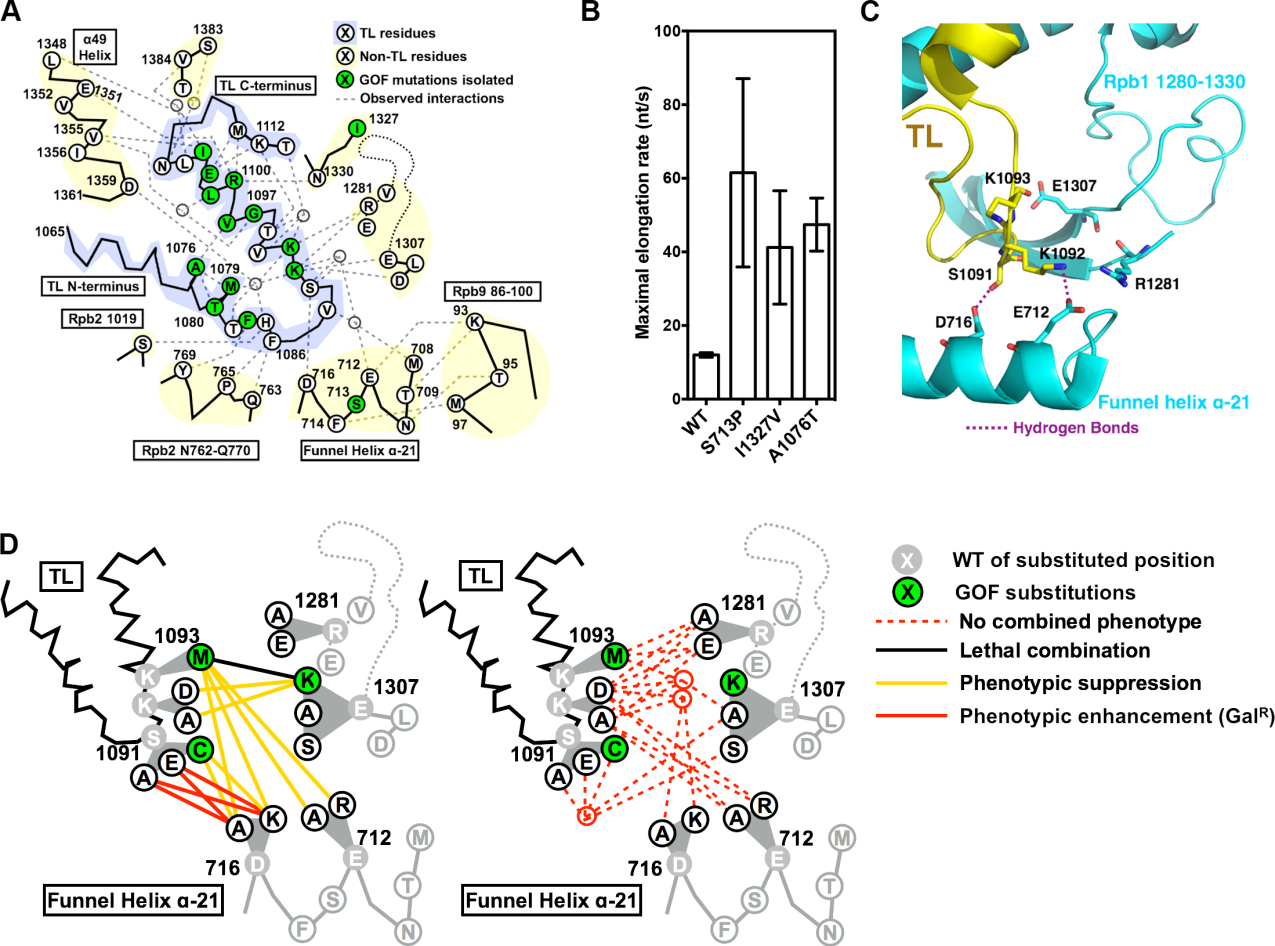
Functional contribution of TL tip and funnel helix α-21 to proper TL dynamics. (A) Observed and predicted interactions between TL and TL-proximal domains. TL schematic is shown with residues identified by single-letter amino acid code and positions of interest annotated. Positions of GOF mutants isolated in our screen, along with the positions for a subset of previously isolated TL-proximal GOF mutants, are color coded in green. Observed TL interactions with other Rpb1 domains from structures or simulation studies are shown as grey dashed lines. (B) Maximal *in vitro* elongation rates (nucleotides/second) of Pol II WT and genetic GOF mutants S713P, I1327V and A1076T. (C) Observed interactions between open TL tip and TL adjacent charged residues (PDB: 5C4X). Funnel Helix refers to the Rpb1 α-21 alpha-helix. (D) Genetic interactions between the TL tip and proximal Rpb1 domains. Schematics of the TL and adjacent domains are shown in lines, with positions of interest shown in single-letter amino acid code. Substituted residues are shown in grey, with substituting amino acids shown in white or green filled circles based on single substitution phenotypes (S8F Fig). Double substitution phenotypes are shown as colored lines connecting the two relevant single substitutions. Some sets of similar interactions were grouped into nodes to reduce complexity in interaction lines.

These changes do not affect the main conclusion of the article, but affect the interpretation of a small subset of D716K or E712A related mutants which are reported in the Results and Discussion. The authors have added an interpretation of transcription-related phenotypes of D716K and its relevant double mutants, and corrected an interpretation of E712A-containing double mutants in comparison to the E712A single substitution mutant. The corrected text is provided below.

## Paragraphs three and four under the subheading “Functional contributions of the TL tip region” in the Results

“Functional interactions among residues can be explored by the similarity between single substitution variants and the phenotypes of double mutants. We first sought evidence that variants in potential TL interaction partners could confer similar GOF or LOF phenotypes. In the simulation, K1092 switched interaction partners between two funnel helix residues D716 and E712 [18], and other charged residues were either observed or simulated to interact with S1091, K1092 or K1093 (Fig 5A). Therefore, we constructed a panel of mutants in the residues D716, E712, R1281, E1307, and D1309 for phenotypic analyses. Notably, we observed GOF phenotypes (Mn^S^ and MPA^S^) in E1307K but not E1307A, suggesting that E1307K gained an interfering interaction to destabilize the open TL state. In contrast, mutations in the residues E712 and D716 did not confer transcription-related phenotypes.

To further dissect functional relationships, we phenotyped double mutants from potential interaction partners, and observed a number of genetic interactions (Fig 5D, S9 Fig). First, mutations in D716 (D716A/K) suppressed the S1091C and K1093M GOF phenotypes and conferred LOF phenotype (Gal^R^) when combined with other S1091 alleles (D716A/S1091A, D716A/S1091E, D716K/S1091A, D716K/S1091E), although the D716A/K or S1091A/E single substitutions did not confer strong transcription-related phenotypes (Fig 5D, S8F and S9A Figs). These allele-specific genetic interactions suggested that D716A/K and S1091A/E lost putatively redundant interactions that together conferred LOF phenotypes, and loss of D716 interaction(s) might also suppress the putatively gain of interactions in GOF mutants S1091C and K1093M. Second, mutations in E712 (E712A/E712R) did not confer transcription-related phenotypes, but suppressed the K1093M GOF phenotypes (S8F and S9B Figs). Similarly, K1092A/D single substitutions did not confer transcription-related phenotypes, but were able to suppress the E1307K GOF phenotypes (S8F and S9E Figs). This observed epistasis suggested that loss of potential interacting residues (E712 and K1092, respectively) relieved putative gain of interactions in the GOF mutants (K1093M and E1307K, respectively) (discussed above). Taken together, the observed allele-specific and epistatic interactions between TL tip and proximal residues suggest a highly complex genetic network of residues controlling TL dynamics, and illustrate how individual residues might constrain or allow diversification of the TL through evolution.”

## Fourth to last sentence of the third paragraph in the Discussion

“In addition to the three previously identified mutants, we utilized a new set of TL mutants to assess genetic interactions between the TL and the funnel helix α-21, and discover epistasis between K1093M (TL) and phenotypically inert E712A/R (funnel helix), along with multiple allele specific genetic interactions (Fig 5D)”.

## Supporting information

S8 Fig Construction and transcription-related phenotypes of the TL tip and nearby charged residue variants. (A) *x*-*y* plot showing the lack of correlation between helical propensity change and phenotypic score on MPA, a good indicator of altered transcription activity. 120 variants from the TL tip region (top panel) and 104 variants from the same region but excluding V1094 mutants (bottom panel) are shown, with linear regression fit of the data shown in black lines. (B-E) Complementation abilities of TL tip (S1091, K1092, K1093) variants, tip proximal D716 (B), E712 (C), R1281 (D), E1307 (E) variants and the corresponding double mutants were determined by plasmid shuffling assays. (F) Transcription-related phenotypes of TL tip and the TL-proximal charged residue variants. S1091C, K1093M and E1307K confer MPA^S^ phenotypes, and K1093M additionally confers an Spt^-^ phenotype, while others alone don’t confer any strong transcription-related phenotypes.
(TIF)

S9 Fig Genetic interactions of the TL tip and nearby charged residue variants on transcription-related phenotypes. Genetic interactions between tip variants and nearby charged residues D716 (A), E712 (B), R1281 (C) and E1307 (D, E) variants detected by alterations in transcription-related phenotypes. Relevant single-substitution mutant phenotypes from S8 Fig are repeatedly shown for ease of comparison with double mutant phenotypes.
(TIF)

S2 Table Yeast strain genotypes and plasmid descriptions. (DOCX)
