# Supplementary figures and images for: High-Resolution Phenotypic Landscape of the RNA Polymerase II Trigger Loop
Source: PLoS Genet. 2016 Nov 29;12(11):e1006321. doi: 10.1371/journal.pgen.1006321 (PMC5127505; doi:10.1371/journal.pgen.1006321)

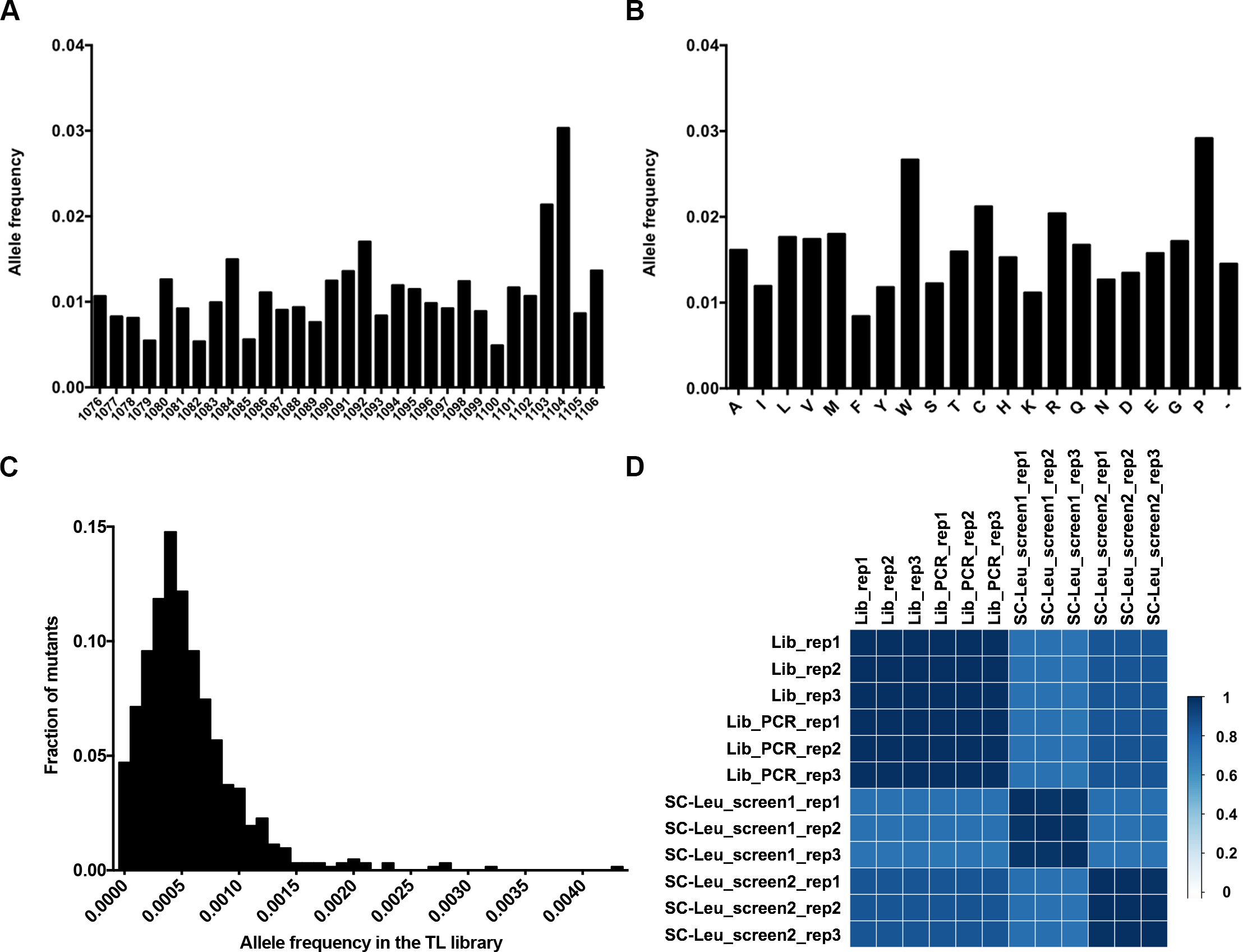

Supplement: S1 Fig — (A) Fraction of TL substitutions at each position of the TL (Rpb1 1076–1106). Allele frequencies were determined by deep sequencing of the TL variant library, and calculated by the number of reads from all the variants at a position divided by the total number of mapped reads. (B) Fraction of TL substitutions for codons encoding specific amino acids. The allele frequency for each substitution was determined by deep sequencing of the TL variant library, calculated by the number of reads for variants substituted at a particular substitution divided by the total number of mapped reads. (C) Distribution of allele frequencies for the detected TL single substitution variants. (D) The TL library is robust to PCR amplification and yeast transformation. Pearson correlation coefficients calculated between different libraries are shown as a heatmap. TL library (Lib), PCR amplified TL library (Lib_PCR) and two yeast pools independently transformed with TL library (SC-Leu_screen1 and SC-Leu_screen2) were amplified and sequenced in triplicate (rep1, rep2 and rep3), and pairwise Pearson correlation analyses were performed between different sequencing libraries. (TIF) [file pgen.1006321.s003.tif]

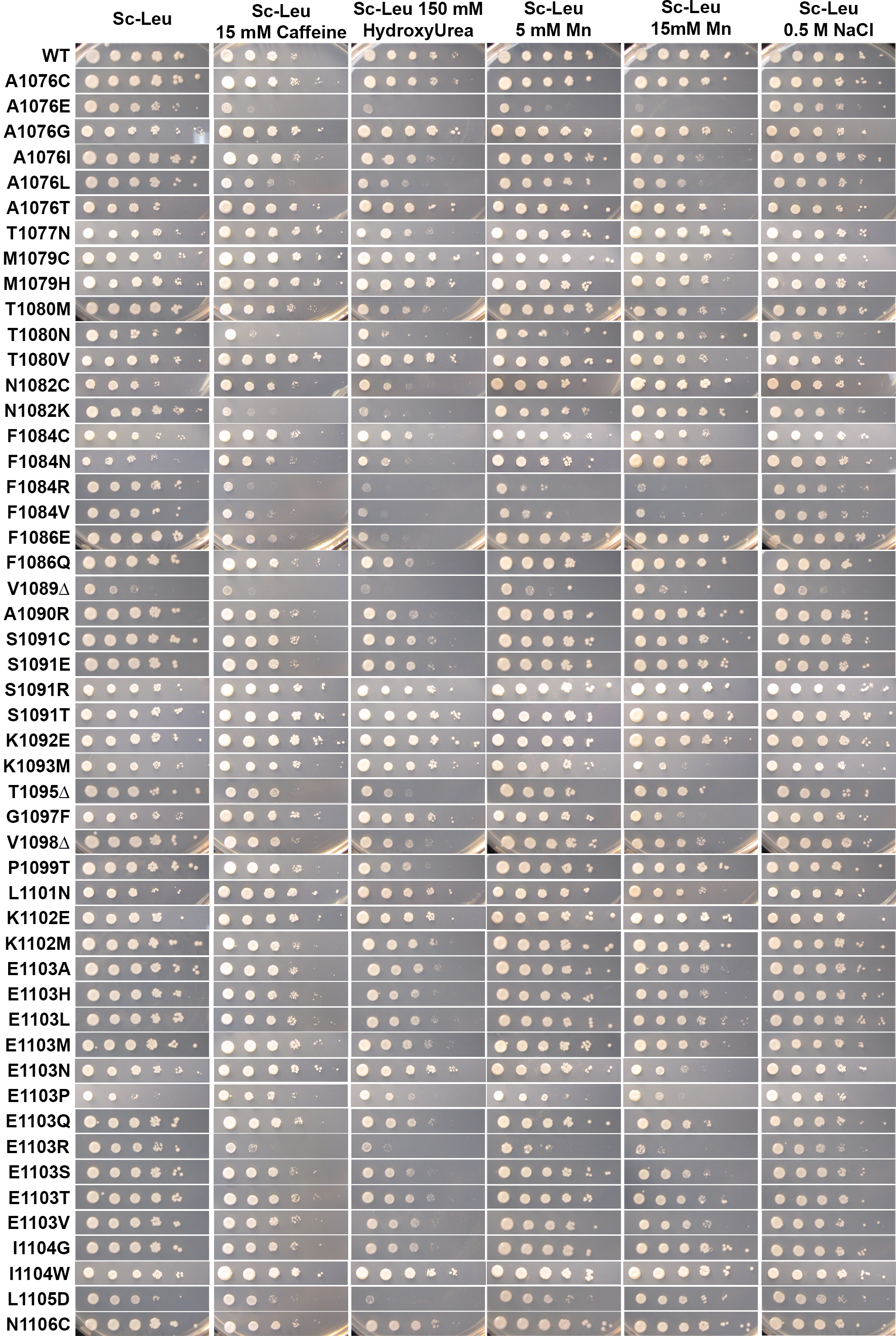

Supplement: S2 Fig — 10-fold serial dilutions of saturated cultures of the 50 TL variants were plated on the indicated conditions, including 15 mM caffeine, 150 mM hydroxyurea, 5 mM Mn2+, 15 mM Mn2+ and 0.5 M NaCl. (TIF) [file pgen.1006321.s004.tif]

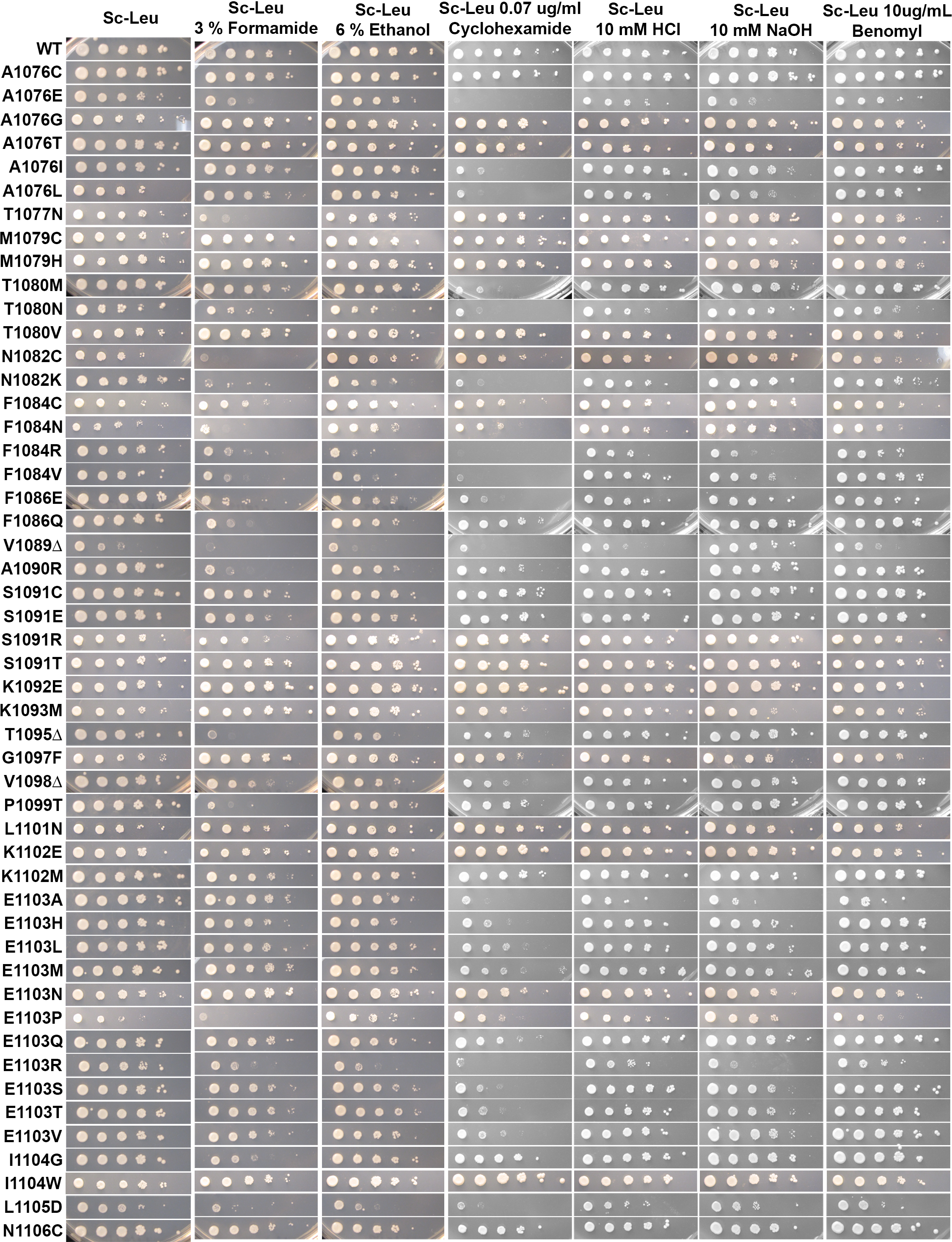

Supplement: S3 Fig — 10-fold serial dilutions of saturated cultures of the 50 TL variants were plated on the indicated conditions, including 3% formamide, 6% ethanol, 0.07 μg/mL cycloheximide, 10 mM HCl, 10 mM NaOH and 10 μg/mL benomyl. (TIF) [file pgen.1006321.s005.tif]

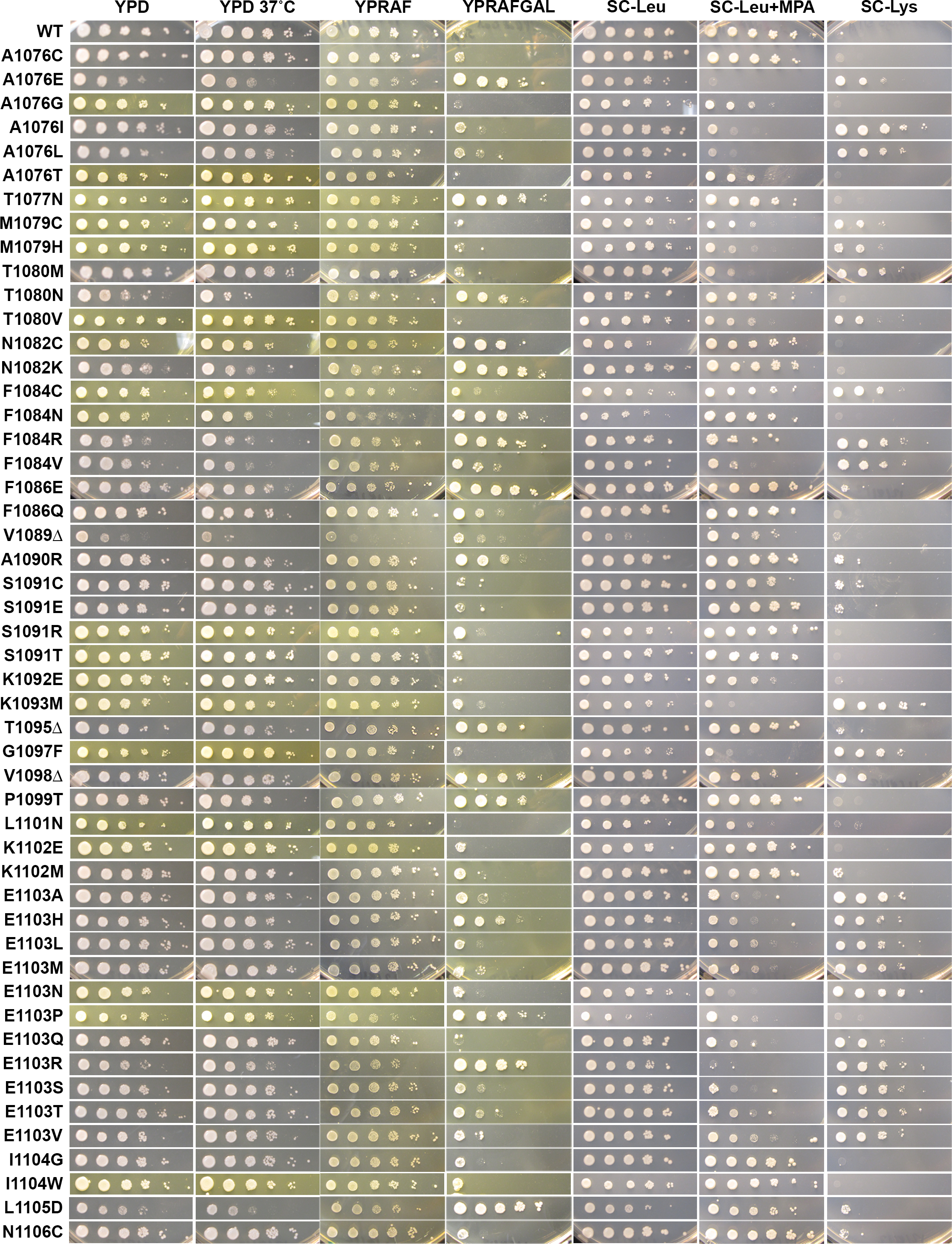

Supplement: S4 Fig — GalR, MPAS and Spt- phenotypes of the 50 TL variants were assessed as a control for the high-throughput phenotyping. (TIF) [file pgen.1006321.s006.tif]

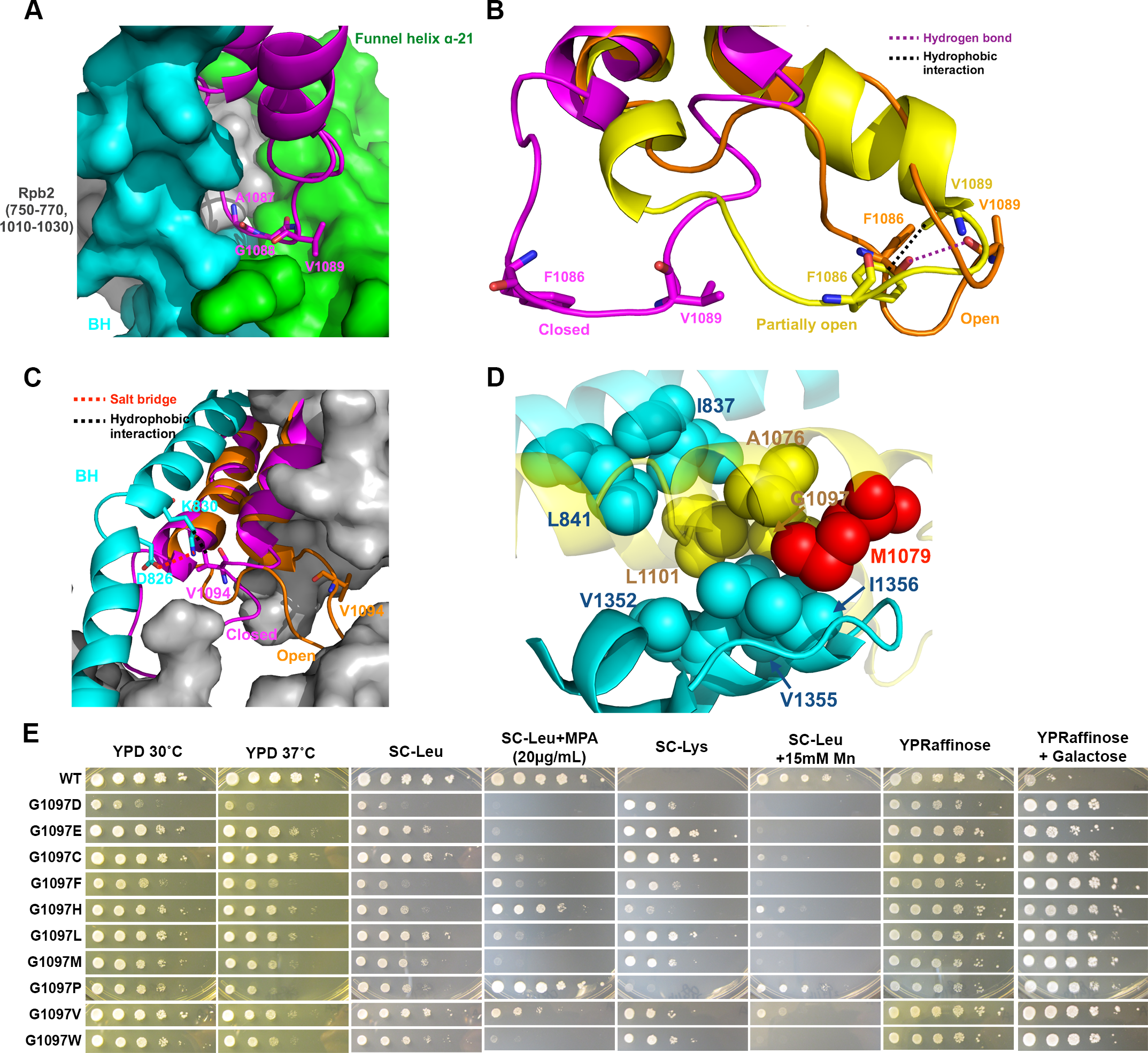

Supplement: S5 Fig — (A) A1087-G1088 linker is highly spatially constrained. The closed TL (magenta) is shown in cartoon (A1087, G1088 in sticks), and TL-proximal domains are shown in surface representation. Rpb2 domains are colored in grey; Bridge Helix (Rpb1 800–860) in cyan; Funnel helix α-21 (Rpb1 700–750) in green. (B) Change of F1086-V1089 interactions in different TL states. V1089 forms a backbone-backbone hydrogen bond with F1086 in the open TL (orange, PDB: 5C4X), but the side chain flips towards the F1086 for a hydrophobic interaction when the TL is in a less open state (yellow, PDB: 5C4J). (C) V1094-K830 interaction in the closed TL state. The charged K830 side chain appears to be neutralized by D826 through a salt bridge interaction, and the neutralized K830 side chain interacts with the V1094 side chain. (D) Observed hydrophobic pocket in the open TL surrounding M1079 (PDB: 5C4J). TL (yellow) and the proximal domains (cyan) are shown in the cartoon representation with the M1079-proximal hydrophobic residues shown in spheres. M1079 is highlighted in red. (E) Transcription-related phenotypes of G1097 variants. (TIF) [file pgen.1006321.s007.tif]

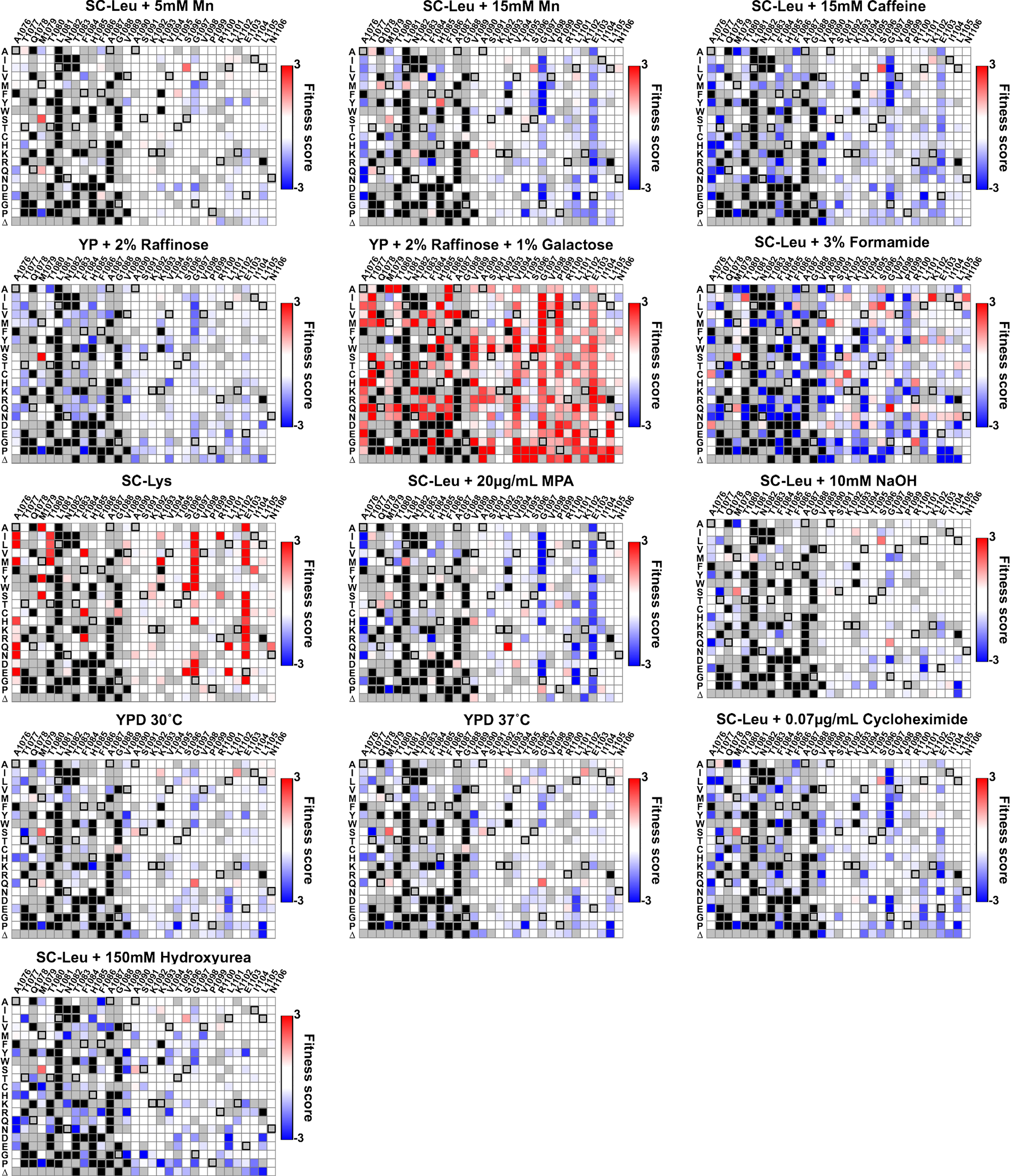

Supplement: S6 Fig — Phenotypic scores of TL single substituted variants under indicated conditions are shown in separate heatmaps. Unavailable data points are shown as filled grey squares. WT residues at indicated positions are outlined in black boxes. Predicted lethal mutants are in filled black squares. (TIF) [file pgen.1006321.s008.tif]

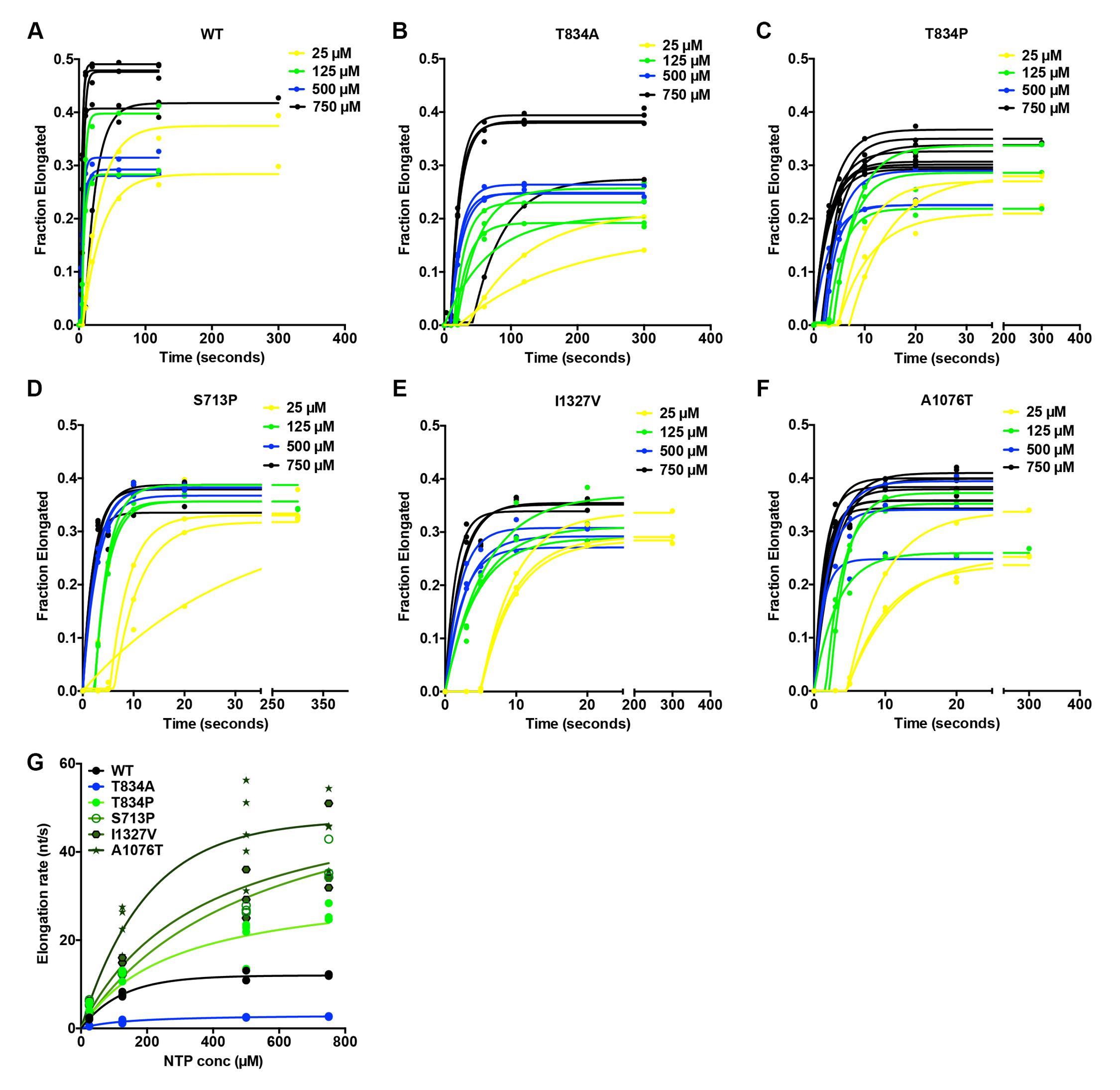

Supplement: S7 Fig — (A-F) Determination of elongation rates at different NTP concentrations for indicated enzymes. Fraction of run-off transcripts by the total (Fraction elongated) was quantified and plotted versus reaction time for indicated Pol II mutants. Lines of different colors indicate the different concentrations of NTPs used. At least three experimental replicates were performed and each replicate was separately curve fitted with non-linear regression (GraphPad Prism 6.0h). (G) Determination of maximal elongation rates for indicated enzymes. Elongation rates (determined from S7A-F Fig) were plotted versus NTP concentrations and curve fitted with non-linear regression (GraphPad Prism 6.0h). (TIF) [file pgen.1006321.s009.tif]

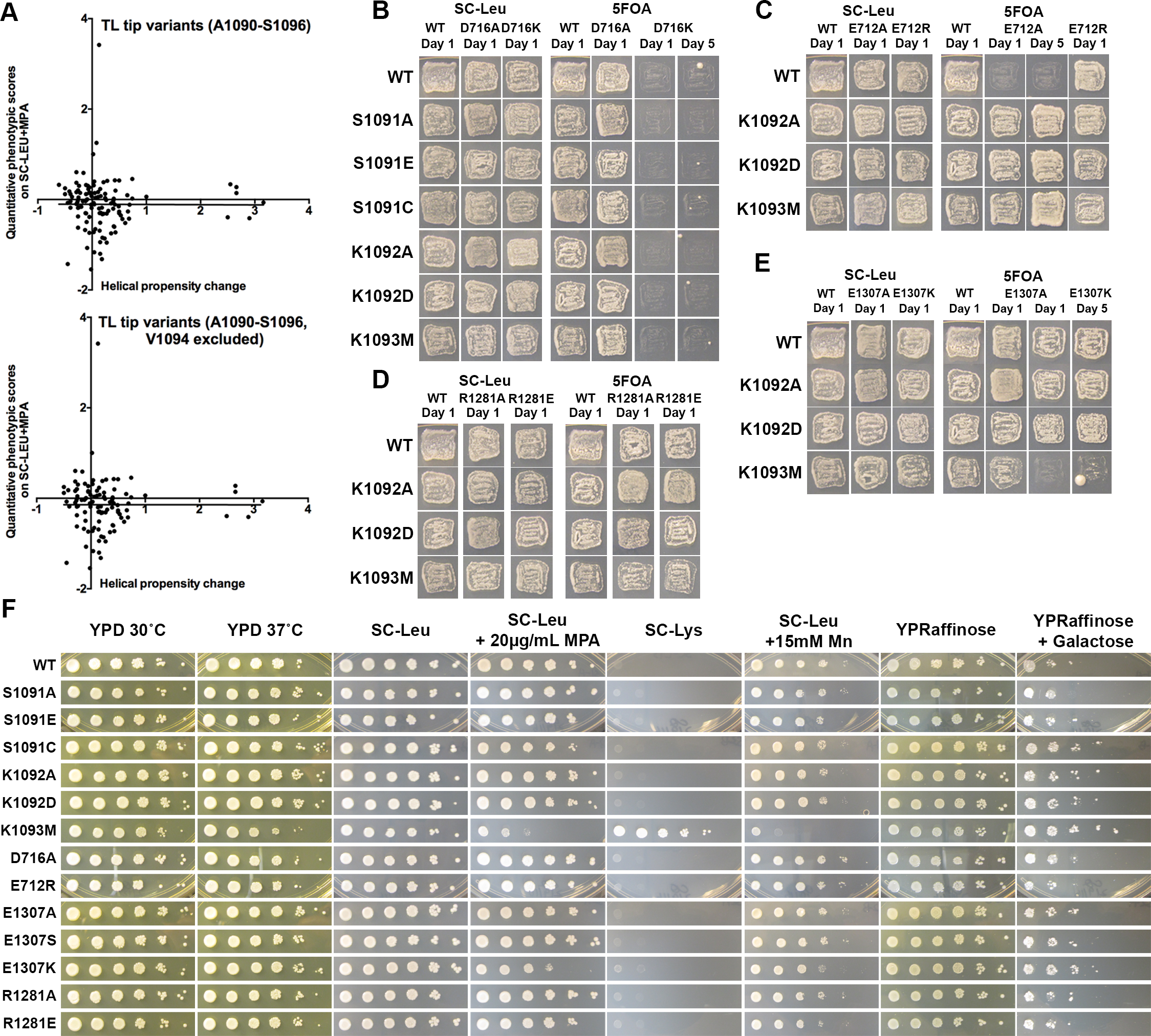

Supplement: S8 Fig — (A) x-y plot showing the lack of correlation between helical propensity change and phenotypic score on MPA, a good indicator of altered transcription activity. 120 variants from the TL tip region (top panel) and 104 variants from the same region but excluding V1094 mutants (bottom panel) are shown, with linear regression fit of the data shown in (some color). (B-E) Complementation abilities of TL tip (S1091, K1092, K1093) variants, tip proximal D716 (B), E712 (C), E1307 (D), R1281 (E) variants and the corresponding double mutants were determined by plasmid shuffling assays. (F) Transcription-related phenotypes of TL tip and the TL-proximal charged residue variants. S1091C, K1093M and E1307K confer MPAS phenotypes, and K1093M additionally confers an Spt- phenotype, while others alone don’t confer any strong transcription-related phenotypes. (TIF) [file pgen.1006321.s010.tif]

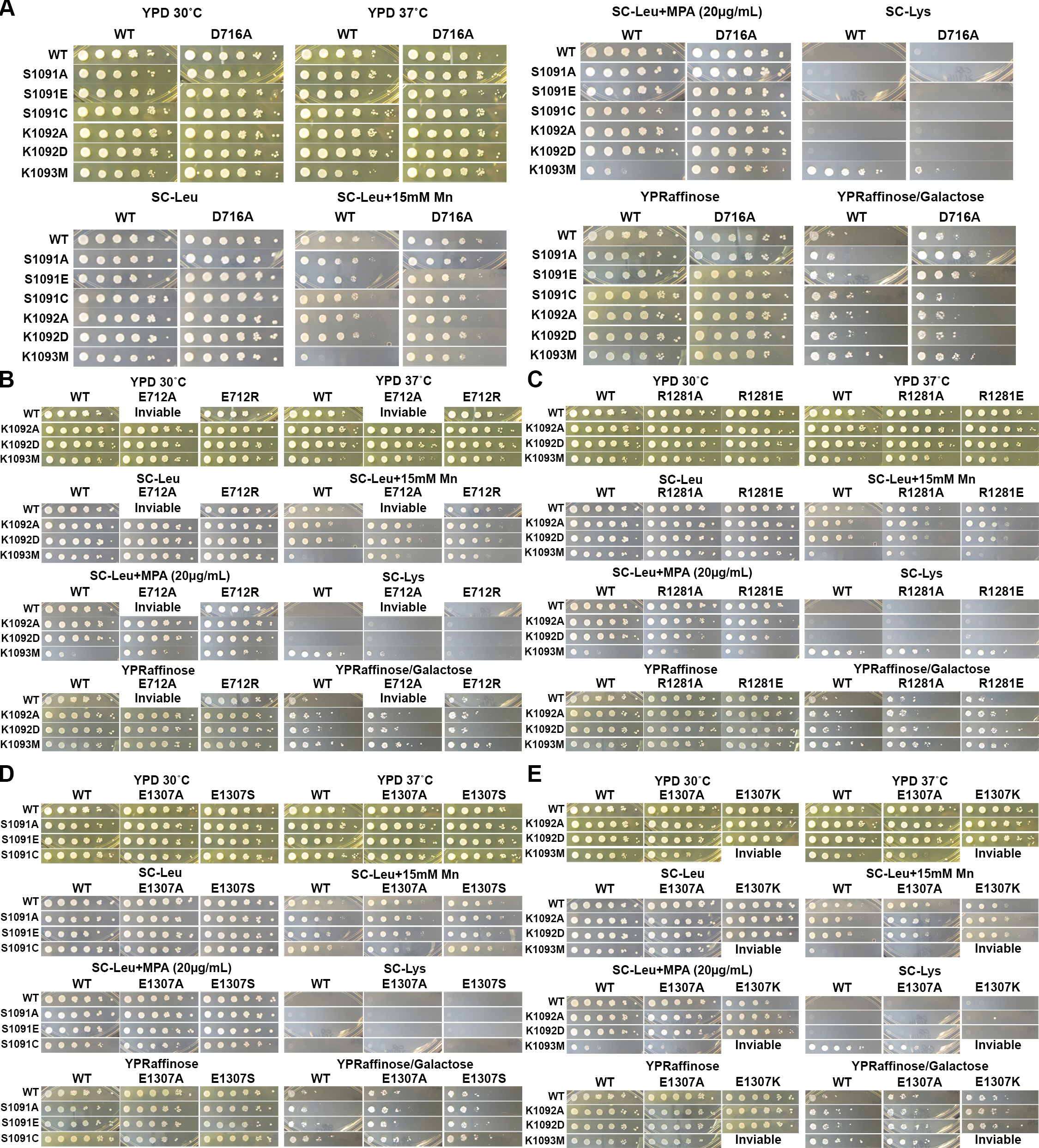

Supplement: S9 Fig — Genetic interactions between tip variants and nearby charged residues D716 (A), E712 (B), R1281 (C) and E1307 (D, E) variants detected by alterations in transcription-related phenotypes. (TIF) [file pgen.1006321.s011.tif]

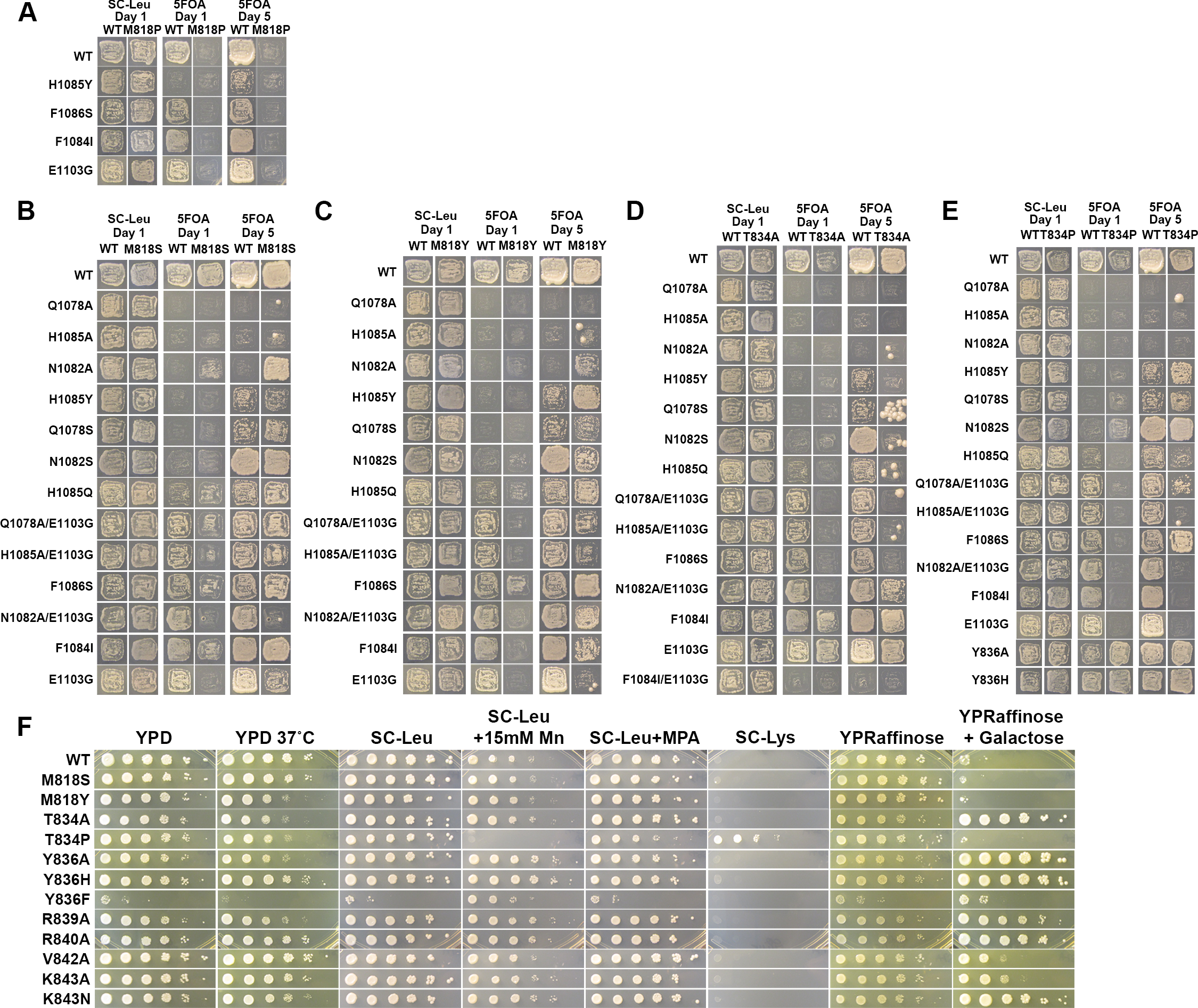

Supplement: S10 Fig — (A-E) Complementation ability of the indicated TL variants, BH single variants M818P (A), M818S (B), M818Y (C), T834A (D), T834P (E) and the corresponding double/triple mutants were determined by plasmid shuffling assays. (F) Transcription-linked phenotypes of BH single-substituted mutants. M818S and M818Y are substitutions in a predicted BH N-terminal hinge; others are substitutions in predicted BH C-terminal hinge positions or additional C-terminal substitutions. (TIF) [file pgen.1006321.s012.tif]

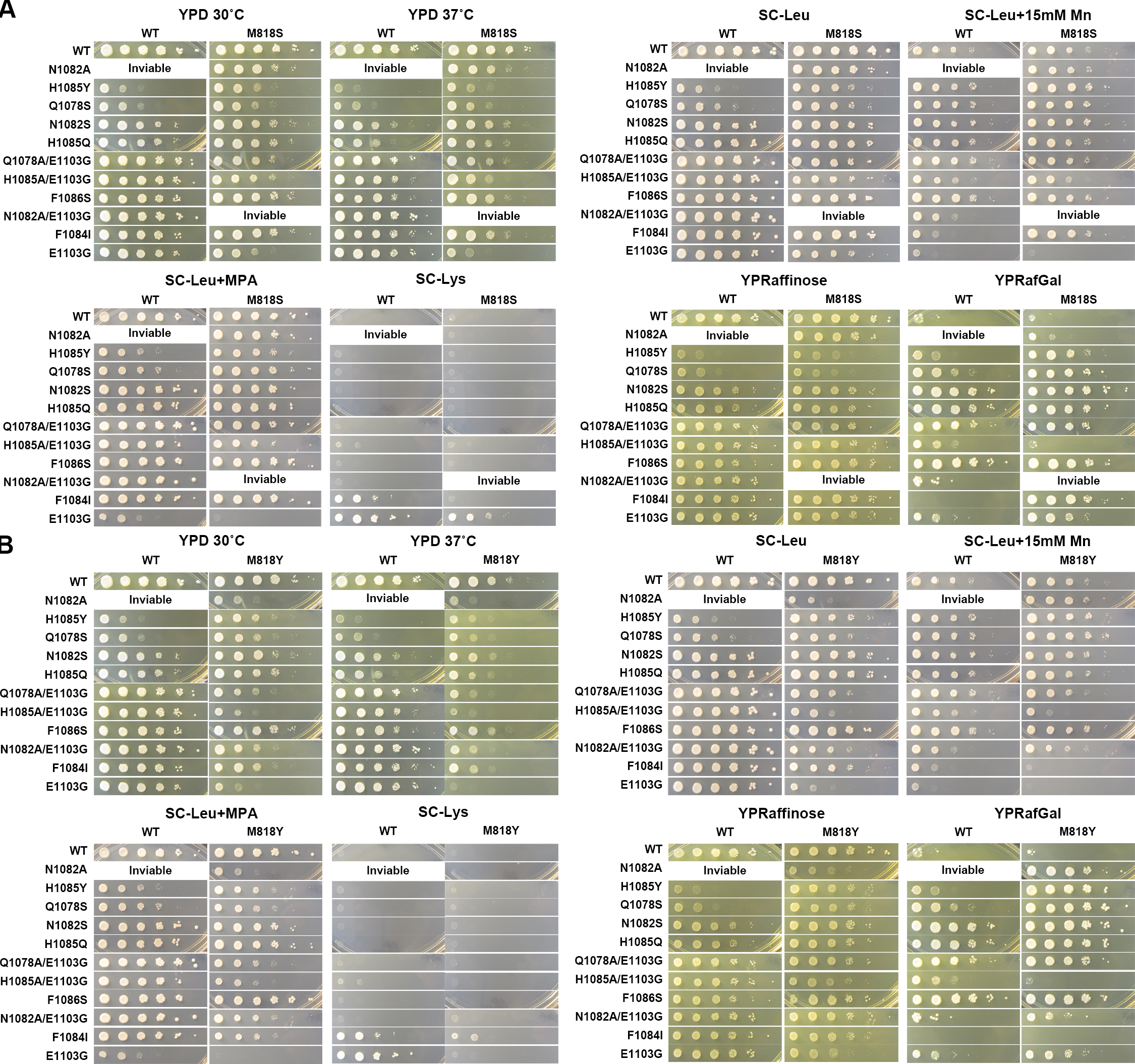

Supplement: S11 Fig — Genetic interactions between TL variants and the BH variants M818S (A), M818Y (B) were assessed by standard plate phenotyping of transcription-related phenotypes. (TIF) [file pgen.1006321.s013.tif]

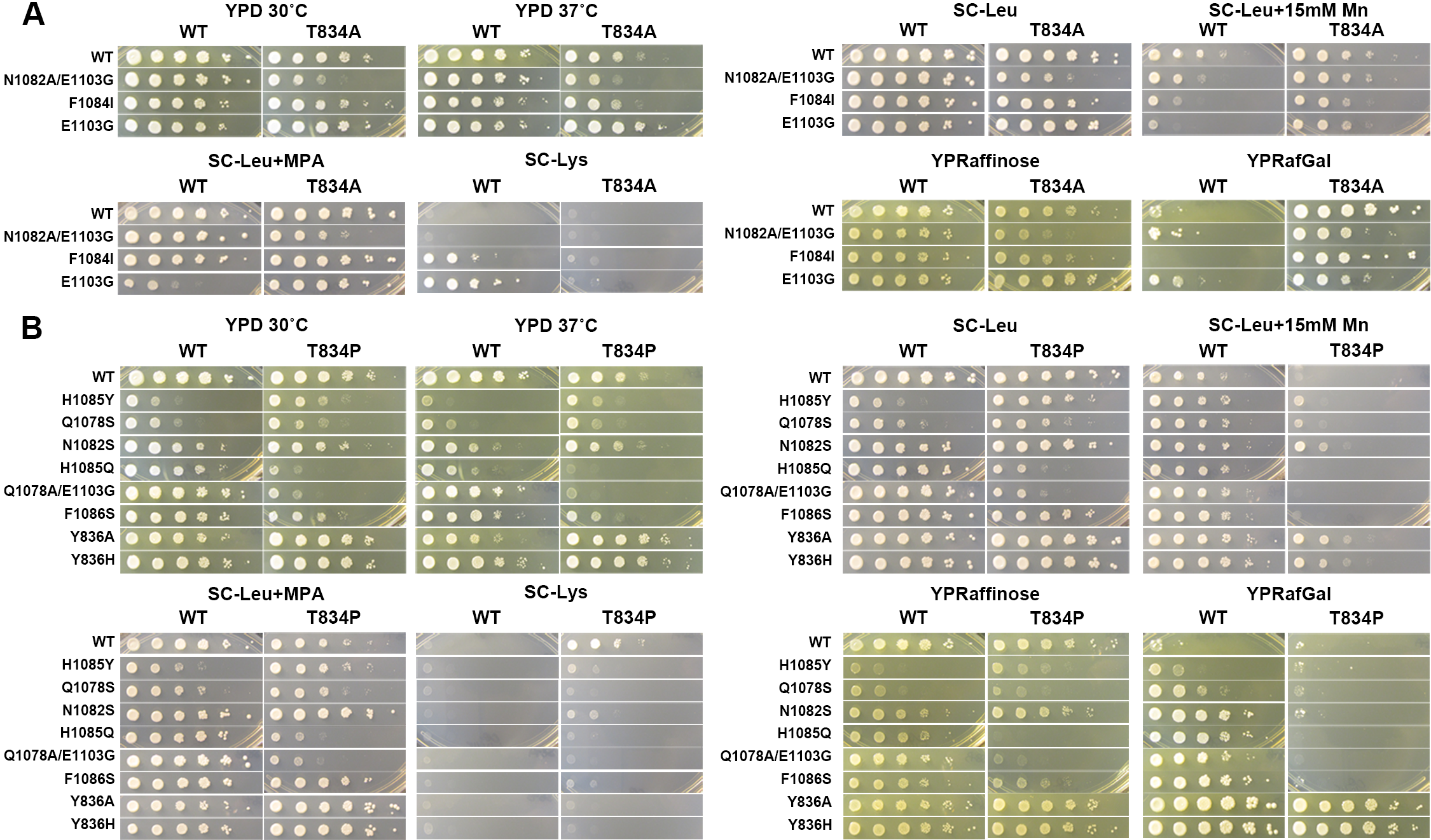

Supplement: S12 Fig — Genetic interactions between TL variants and the BH variants T834A (A), T834P (B) were assessed by standard plate phenotyping of transcription-related phenotypes. Additional genetic interactions between T834P (GOF) and two LOF BH mutants (Y836A) and Y836H are included in (B). (TIF) [file pgen.1006321.s014.tif]
