# Supplementary figures and images for: Correction: High-Resolution Phenotypic Landscape of the RNA Polymerase II Trigger Loop
Source: PLoS Genet. 2018 Jan 3;14(1):e1007158. doi: 10.1371/journal.pgen.1007158 (PMC5751974; doi:10.1371/journal.pgen.1007158)

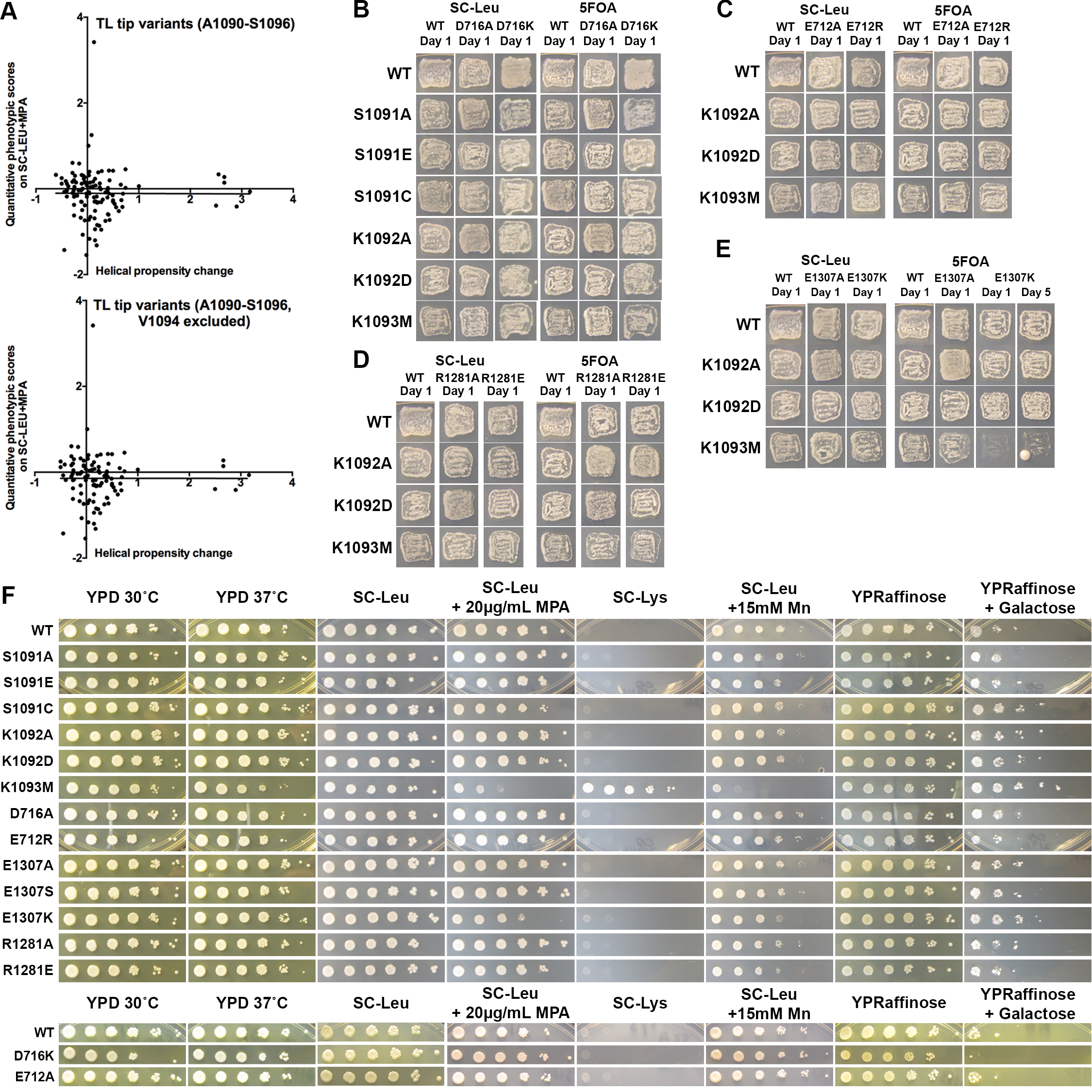

Supplement: S8 Fig — (A) x-y plot showing the lack of correlation between helical propensity change and phenotypic score on MPA, a good indicator of altered transcription activity. 120 variants from the TL tip region (top panel) and 104 variants from the same region but excluding V1094 mutants (bottom panel) are shown, with linear regression fit of the data shown in black lines. (B-E) Complementation abilities of TL tip (S1091, K1092, K1093) variants, tip proximal D716 (B), E712 (C), R1281 (D), E1307 (E) variants and the corresponding double mutants were determined by plasmid shuffling assays. (F) Transcription-related phenotypes of TL tip and the TL-proximal charged residue variants. S1091C, K1093M and E1307K confer MPAS phenotypes, and K1093M additionally confers an Spt- phenotype, while others alone don’t confer any strong transcription-related phenotypes. (TIF) [file pgen.1007158.s001.tif]

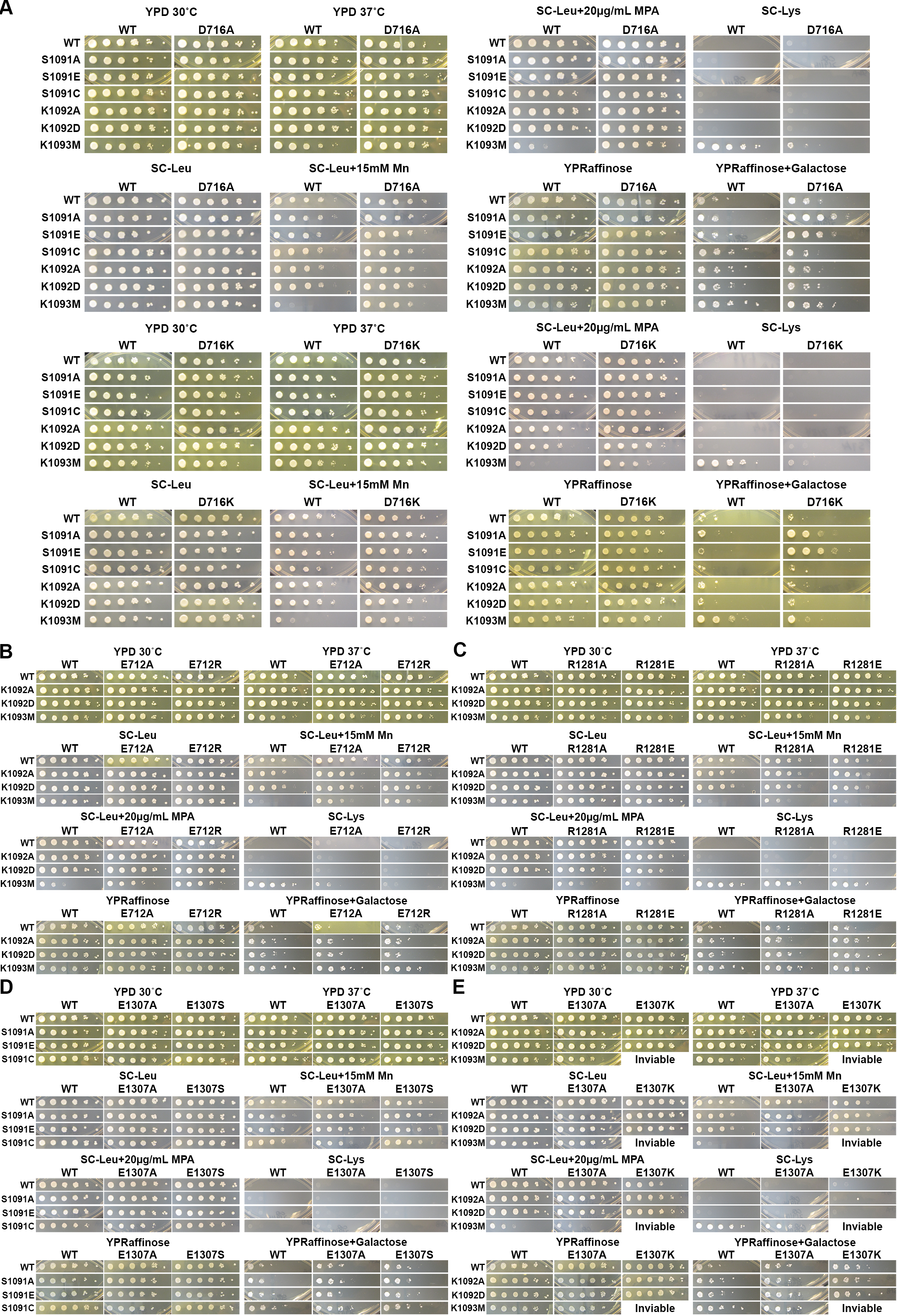

Supplement: S9 Fig — Genetic interactions between tip variants and nearby charged residues D716 (A), E712 (B), R1281 (C) and E1307 (D, E) variants detected by alterations in transcription-related phenotypes. Relevant single-substitution mutant phenotypes from S8 Fig are repeatedly shown for ease of comparison with double mutant phenotypes. (TIF) [file pgen.1007158.s002.tif]
