# Supplementary material for: Correction: High-Resolution Phenotypic Landscape of the RNA Polymerase II Trigger Loop
Source: PLoS Genet. 2018 Jan 3;14(1):e1007158. doi: 10.1371/journal.pgen.1007158 (PMC5751974; doi:10.1371/journal.pgen.1007158)
Supplement: S2 Table — (DOCX) [file pgen.1007158.s003.docx]

S2 Table: Yeast strain genotypes and plasmid descriptions.

S2 Table Summary: This table includes two tabs: "Strains" and "Plasmids". Most of the reported rpb1 mutants are analyzed in the CKY283 background via plasmid shuffling strategy (described in the main text). For any given analyzed rpb1 mutant, the mutation was verified by sequencing, and the "substituting Domain", "Viability" and the "Derivation of the plasmid" (either from "Sub-cloned from previous screening" or "site-directed mutagenesis") are reported. Reference for previously reported plasmids are noted in the last column.

S2 Table Strains:

| **Strain Number** | **Genotype** | **Reference** |
| --- | --- | --- |
| CKY283 | *MAT***a** *ura3-52 his3∆200 leu2∆1 or ∆0 trp1∆63 met15∆0 lys2-128∂ gal10∆56 rpb1∆::CLONATMX RPB3::TAP::KlacTRP1* [pRP112 *RPB1 URA3 CEN*] | Kaplan et al, Plos Genetics, 2012 |
| CKY1343 | *ura3-52 his3∆200 leu2∆1 or ∆0 trp1∆63 met15∆0 lys2-128∂ gal10∆56 rpb1∆::CLONATMX RPB3::TAP::KlacTRP1 pmr1∆::hphmx [pRP112 RPB1 CEN URA3]* | This study |

S2 Table Plasmids:

| **Mutant** | **Plasmid number** | **Substituting Domain** | **Viability** | **Derivation of the mutants** | **Reference** |
| --- | --- | --- | --- | --- | --- |
| WT (URA3) | pCK250 (pRP112) | N.A. | Viable | N.A. | [1] |
| WT | pCK859 | N.A. | Viable | N.A. | [1] |
| TL∆ Rpb1 | pCK892 | TL | Inviable | N.A. | [3] |
| A1076T | pCK968 | TL | Viable | Sub-cloned from previous screening | [3] |
| Q1078A | pCK935 | TL | Inviable | Site-directed mutagenesis | [2] |
| Q1078S | pCK863 | TL | Viable | Site-directed mutagenesis | [2] |
| M1079R | pCK872 | TL | Viable | Site-directed mutagenesis | [2], [3] |
| N1082A | pCK885 | TL | Inviable | Site-directed mutagenesis | [2] |
| N1082S | pCK886 | TL | Viable | Site-directed mutagenesis | [2], [3] |
| F1084I | pCK955 | TL | Viable | Sub-cloned from previous screening | [1], [2], [3] |
| H1085A | pCK861 | TL | Inviable | Site-directed mutagenesis | [1], [2] |
| H1085Q | pCK887 | TL | Viable | Site-directed mutagenesis | [2], [3] |
| H1085Y | pCK870 | TL | Viable | Site-directed mutagenesis | [1], [2] |
| F1086S | pCK871 | TL | Viable | Sub-cloned from previous screening | [1], [2], [3] |
| G1097D (with silent mutation) | pCK867 | TL | Viable | Sub-cloned from previous screening | [1], [2] |
| L1101S | pCK864 | TL | Viable | Sub-cloned from previous screening | [1], [2], [3] |
| E1103G | pCK960 | TL | Viable | Sub-cloned from previous screening | [1], [2], [3], [4], [5] |
| Q1078A/E1103G | pCK947 | TL | Viable | Site-directed mutagenesis | [2] |
| N1082A/E1103G | pCK897 | TL | Viable | Site-directed mutagenesis | [2] |
| F1084I/E1103G | pCK952 | TL | Inviable | Site-directed mutagenesis | [2] |
| H1085A/E1103G | pCK899 | TL | Viable | Site-directed mutagenesis | [2] |
| S713P | pCK866 | Funnel helix | Viable | Sub-cloned from previous screening | [3] |
| I1327V | pCK610 | Other Rpb1 domains | Viable | Sub-cloned from previous screening | [3] |
| A1087G | pCK1730 | TL | Inviable | Site-directed mutagenesis | This study |
| G1088A | pCK1731 | TL | Inviable | Site-directed mutagenesis | This study |
| A1087G/G1088A | pCK1732 | TL | Inviable | Site-directed mutagenesis | This study |
| A1087V | pCK1733 | TL | Viable | Site-directed mutagenesis | This study |
| G1088V | pCK1734 | TL | Inviable | Site-directed mutagenesis | This study |
| L1081M | pCK1735 | TL | Viable | Site-directed mutagenesis | This study |
| L1081P | pCK1736 | TL | Inviable | Site-directed mutagenesis | This study |
| H1085L | pCK1748 | TL | Viable | Site-directed mutagenesis | This study |
| M818P | pCK1089 | BH | Inviable | Site-directed mutagenesis | This study |
| M818S | pCK1165 | BH | Viable | Site-directed mutagenesis | This study |
| M818Y | pCK1164 | BH | Viable | Site-directed mutagenesis | This study |
| T834A | pCK910 | BH | Viable | Site-directed mutagenesis | This study |
| T834P | pCK1087 | BH | Viable | Site-directed mutagenesis | This study |
| Y836A | pCK1899 | BH | Viable | Site-directed mutagenesis | This study |
| Y836H | pCK1901 | BH | Viable | Site-directed mutagenesis | This study |
| Y836F | pCK1903 | BH | Viable | Site-directed mutagenesis | This study |
| R839A | pCK1904 | BH | Viable | Site-directed mutagenesis | This study |
| R840A | pCK1905 | BH | Viable | Site-directed mutagenesis | This study |
| V842A | pCK1906 | BH | Viable | Site-directed mutagenesis | This study |
| K843A | pCK1907 | BH | Viable | Site-directed mutagenesis | This study |
| K843N | pCK1908 | BH | Viable | Site-directed mutagenesis | This study |
| M818P/H1085Y | pCK1117 | BH/TL | Inviable | Site-directed mutagenesis | This study |
| M818P/F1086S | pCK1118 | BH/TL | Inviable | Site-directed mutagenesis | This study |
| M818P/F1084I | pCK1119 | BH/TL | Inviable | Site-directed mutagenesis | This study |
| M818P/E1103G | pCK1120 | BH/TL | Inviable | Site-directed mutagenesis | This study |
| M818S/Q1078A | pCK1868 | BH/TL | Inviable | Site-directed mutagenesis | This study |
| M818S/H1085A | pCK1869 | BH/TL | Viable | Site-directed mutagenesis | This study |
| M818S/N1082A | pCK1870 | BH/TL | Viable | Site-directed mutagenesis | This study |
| M818S/H1085Y | pCK1871 | BH/TL | Viable | Site-directed mutagenesis | This study |
| M818S/Q1078S | pCK1872 | BH/TL | Viable | Site-directed mutagenesis | This study |
| M818S/N1082S | pCK1873 | BH/TL | Viable | Site-directed mutagenesis | This study |
| M818S/H1085Q | pCK1874 | BH/TL | Viable | Site-directed mutagenesis | This study |
| M818S/Q1078A/E1103G | pCK1875 | BH/TL | Viable | Site-directed mutagenesis | This study |
| M818S/H1085A/E1103G | pCK1876 | BH/TL | Viable | Site-directed mutagenesis | This study |
| M818S/F1086S | pCK1877 | BH/TL | Viable | Site-directed mutagenesis | This study |
| M818S/N1082A/E1103G | pCK1878 | BH/TL | Inviable | Site-directed mutagenesis | This study |
| M818S/F1084I | pCK1879 | BH/TL | Viable | Site-directed mutagenesis | This study |
| M818S/E1103G | pCK1880 | BH/TL | Viable | Site-directed mutagenesis | This study |
| M818Y/Q1078A | pCK1881 | BH/TL | Inviable | Site-directed mutagenesis | This study |
| M818Y/H1085A | pCK1882 | BH/TL | Viable | Site-directed mutagenesis | This study |
| M818Y/N1082A | pCK1883 | BH/TL | Viable | Site-directed mutagenesis | This study |
| M818Y/H1085Y | pCK1166 | BH/TL | Viable | Site-directed mutagenesis | This study |
| M818Y/Q1078S | pCK1884 | BH/TL | Viable | Site-directed mutagenesis | This study |
| M818Y/N1082S | pCK1885 | BH/TL | Viable | Site-directed mutagenesis | This study |
| M818Y/H1085Q | pCK1886 | BH/TL | Viable | Site-directed mutagenesis | This study |
| M818Y/Q1078A/E1103G | pCK1887 | BH/TL | Viable | Site-directed mutagenesis | This study |
| M818Y/H1085A/E1103G | pCK1888 | BH/TL | Viable | Site-directed mutagenesis | This study |
| M818Y/F1086S | pCK1167 | BH/TL | Viable | Site-directed mutagenesis | This study |
| M818Y/N1082A/E1103G | pCK1889 | BH/TL | Viable | Site-directed mutagenesis | This study |
| M818Y/F1084I | pCK1169 | BH/TL | Viable | Site-directed mutagenesis | This study |
| M818Y/E1103G | pCK1168 | BH/TL | Viable | Site-directed mutagenesis | This study |
| T834A/Q1078A | pCK1403 | BH/TL | Inviable | Site-directed mutagenesis | This study |
| T834A/H1085A | pCK1399 | BH/TL | Inviable | Site-directed mutagenesis | This study |
| T834A/N1082A | pCK1411 | BH/TL | Inviable | Site-directed mutagenesis | This study |
| T834A/H1085Y | pCK1408 | BH/TL | Inviable | Site-directed mutagenesis | This study |
| T834A/Q1078S | pCK1400 | BH/TL | Inviable | Site-directed mutagenesis | This study |
| T834A/N1082S | pCK1401 | BH/TL | Inviable | Site-directed mutagenesis | This study |
| T834A/H1085Q | pCK1402 | BH/TL | Inviable | Site-directed mutagenesis | This study |
| T834A/Q1078A/E1103G | pCK1406 | BH/TL | Inviable | Site-directed mutagenesis | This study |
| T834A/H1085A/E1103G | pCK1405 | BH/TL | Inviable | Site-directed mutagenesis | This study |
| T834A/F1086S | pCK1407 | BH/TL | Inviable | Site-directed mutagenesis | This study |
| T834A/N1082A/E1103G | pCK1404 | BH/TL | Viable | Site-directed mutagenesis | This study |
| T834A/F1084I | pCK1409 | BH/TL | Viable | Site-directed mutagenesis | This study |
| T834A/E1103G | pCK1410 | BH/TL | Viable | Site-directed mutagenesis | This study |
| T834A/F1084I/E1103G | pCK1112 | BH/TL | Inviable | Site-directed mutagenesis | This study |
| T834P/Q1078A | pCK1890 | BH/TL | Inviable | Site-directed mutagenesis | This study |
| T834P/H1085A | pCK1891 | BH/TL | Inviable | Site-directed mutagenesis | This study |
| T834P/N1082A | pCK1892 | BH/TL | Inviable | Site-directed mutagenesis | This study |
| T834P/H1085Y | pCK1113 | BH/TL | Viable | Site-directed mutagenesis | This study |
| T834P/Q1078S | pCK1893 | BH/TL | Viable | Site-directed mutagenesis | This study |
| T834P/N1082S | pCK1894 | BH/TL | Viable | Site-directed mutagenesis | This study |
| T834P/H1085Q | pCK1895 | BH/TL | Viable | Site-directed mutagenesis | This study |
| T834P/Q1078A/E1103G | pCK1896 | BH/TL | Viable | Site-directed mutagenesis | This study |
| T834P/H1085A/E1103G | pCK1897 | BH/TL | Inviable | Site-directed mutagenesis | This study |
| T834P/F1086S | pCK1114 | BH/TL | Viable | Site-directed mutagenesis | This study |
| T834P/N1082A/E1103G | pCK1898 | BH/TL | Inviable | Site-directed mutagenesis | This study |
| T834P/F1084I | pCK1115 | BH/TL | Inviable | Site-directed mutagenesis | This study |
| T834P/E1103G | pCK1116 | BH/TL | Inviable | Site-directed mutagenesis | This study |
| T834P/Y836A | pCK1900 | BH | Viable | Site-directed mutagenesis | This study |
| T834P/Y836H | pCK1902 | BH | Viable | Site-directed mutagenesis | This study |
| G1097E | pCK1737 | TL | Viable | Site-directed mutagenesis | This study |
| G1097D | pCK1738 | TL | Viable | Site-directed mutagenesis | This study |
| S1091A | pCK1749 | TL | Viable | Site-directed mutagenesis | This study |
| S1091E | pCK1756 | TL | Viable | Site-directed mutagenesis | This study |
| S1091C | pCK1750 | TL | Viable | Site-directed mutagenesis | This study |
| K1092A | pCK1751 | TL | Viable | Site-directed mutagenesis | This study |
| K1092D | pCK1752 | TL | Viable | Site-directed mutagenesis | This study |
| K1093M | pCK1755 | TL | Viable | Site-directed mutagenesis | This study |
| D716A | pCK1747 | Funnel helix | Viable | Site-directed mutagenesis | This study |
| D716K | pCK2221 | Funnel helix | Viable | Site-directed mutagenesis | This study |
| E712A | pCK1740 | Funnel helix | Viable | Site-directed mutagenesis | This study |
| E712R | pCK1741 | Funnel helix | Viable | Site-directed mutagenesis | This study |
| E1307A | pCK1742 | TL tip proximal | Viable | Site-directed mutagenesis | This study |
| E1307S | pCK1743 | TL tip proximal | Viable | Site-directed mutagenesis | This study |
| E1307K | pCK1744 | TL tip proximal | Viable | Site-directed mutagenesis | This study |
| R1281A | pCK1745 | TL tip proximal | Viable | Site-directed mutagenesis | This study |
| R1281E | pCK1746 | TL tip proximal | Viable | Site-directed mutagenesis | This study |
| D716A/S1091A | pCK1757 | Funnel helix/TL | Viable | Site-directed mutagenesis | This study |
| D716A/S1091E | pCK1759 | Funnel helix/TL | Viable | Site-directed mutagenesis | This study |
| D716A/S1091C | pCK1761 | Funnel helix/TL | Viable | Site-directed mutagenesis | This study |
| D716A/K1092A | pCK1769 | Funnel helix/TL | Viable | Site-directed mutagenesis | This study |
| D716A/K1092D | pCK1777 | Funnel helix/TL | Viable | Site-directed mutagenesis | This study |
| D716A/K1093M | pCK1785 | Funnel helix/TL | Viable | Site-directed mutagenesis | This study |
| D716K/S1091A | pCK2224 | Funnel helix/TL | Viable | Site-directed mutagenesis | This study |
| D716K/S1091E | pCK2239 | Funnel helix/TL | Viable | Site-directed mutagenesis | This study |
| D716K/S1091C | pCK2225 | Funnel helix/TL | Viable | Site-directed mutagenesis | This study |
| D716K/K1092A | pCK2226 | Funnel helix/TL | Viable | Site-directed mutagenesis | This study |
| D716K/K1092D | pCK2227 | Funnel helix/TL | Viable | Site-directed mutagenesis | This study |
| D716K/K1093M | pCK2228 | Funnel helix/TL | Viable | Site-directed mutagenesis | This study |
| E712A/K1092A | pCK1771 | Funnel helix/TL | Viable | Site-directed mutagenesis | This study |
| E712A/K1092D | pCK1779 | Funnel helix/TL | Viable | Site-directed mutagenesis | This study |
| E712A/K1093M | pCK1787 | Funnel helix/TL | Viable | Site-directed mutagenesis | This study |
| E712R/K1092A | pCK1772 | Funnel helix/TL | Viable | Site-directed mutagenesis | This study |
| E712R/K1092D | pCK1780 | Funnel helix/TL | Viable | Site-directed mutagenesis | This study |
| E712R/K1093M | pCK1788 | Funnel helix/TL | Viable | Site-directed mutagenesis | This study |
| E1307A/K1092A | pCK1775 | TL tip proximal/TL | Viable | Site-directed mutagenesis | This study |
| E1307A/K1092D | pCK1783 | TL tip proximal/TL | Viable | Site-directed mutagenesis | This study |
| E1307A/K1093M | pCK1791 | TL tip proximal/TL | Viable | Site-directed mutagenesis | This study |
| E1307K/K1092A | pCK1776 | TL tip proximal/TL | Viable | Site-directed mutagenesis | This study |
| E1307K/K1092D | pCK1784 | TL tip proximal/TL | Viable | Site-directed mutagenesis | This study |
| E1307K/K1093M | pCK1792 | TL tip proximal/TL | Inviable | Site-directed mutagenesis | This study |
| R1281A/K1092A | pCK1773 | TL tip proximal/TL | Viable | Site-directed mutagenesis | This study |
| R1281A/K1092D | pCK1781 | TL tip proximal/TL | Viable | Site-directed mutagenesis | This study |
| R1281A/K1093M | pCK1789 | TL tip proximal/TL | Viable | Site-directed mutagenesis | This study |
| R1281E/K1092A | pCK1774 | TL tip proximal/TL | Viable | Site-directed mutagenesis | This study |
| R1281E/K1092D | pCK1782 | TL tip proximal/TL | Viable | Site-directed mutagenesis | This study |
| R1281E/K1093M | pCK1790 | TL tip proximal/TL | Viable | Site-directed mutagenesis | This study |

| Reference: |
| --- |
| [1] Kaplan, C. D., K. M. Larsson and R. D. Kornberg (2008). "The RNA polymerase II trigger loop functions in substrate selection and is directly targeted by alpha-amanitin." Mol Cell 30(5): 547-556. |
| [2] Kaplan, C. D., H. Jin, I. L. Zhang and A. Belyanin (2012). "Dissection of Pol II trigger loop function and Pol II activity-dependent control of start site selection in vivo." PLoS Genet 8(4): e1002627. |
| [3] Braberg, H., H. Jin, E. A. Moehle, Y. A. Chan, S. Wang, M. Shales, J. J. Benschop, J. H. Morris, C. Qiu, F. Hu, L. K. Tang, J. S. Fraser, F. C. Holstege, P. Hieter, C. Guthrie, C. D. Kaplan and N. J. Krogan (2013). "From structure to systems: high-resolution, quantitative genetic analysis of RNA polymerase II." Cell 154(4): 775-788. |
| [4] Malagon, F., M. L. Kireeva, B. K. Shafer, L. Lubkowska, M. Kashlev and J. N. Strathern (2006). "Mutations in the Saccharomyces cerevisiae RPB1 gene conferring hypersensitivity to 6-azauracil." Genetics 172(4): 2201-2209. |
| [5] Kireeva, M. L., Y. A. Nedialkov, G. H. Cremona, Y. A. Purtov, L. Lubkowska, F. Malagon, Z. F. Burton, J. N. Strathern and M. Kashlev (2008). "Transient reversal of RNA polymerase II active site closing controls fidelity of transcription elongation." Mol Cell 30(5): 557-566. |
